# The gasdermin family: emerging therapeutic targets in diseases

**DOI:** 10.1038/s41392-024-01801-8

**Published:** 2024-04-08

**Authors:** Chenglong Zhu, Sheng Xu, Ruoyu Jiang, Yizhi Yu, Jinjun Bian, Zui Zou

**Affiliations:** 1 Faculty of Anesthesiology, Changhai Hospital, Naval Medical University, Shanghai, 200433 China; 2 School of Anesthesiology, Naval Medical University, Shanghai, 200433 China; 3 National Key Laboratory of Immunity & Inflammation, Naval Medical University, Shanghai, 200433 China; 4 Department of Biochemistry and Molecular Biology, College of Basic Medical Sciences, Naval Medical University, Shanghai, 200433 China

**Keywords:** Immunological disorders, Cell biology, Diseases

## Abstract

The gasdermin (GSDM) family has garnered significant attention for its pivotal role in immunity and disease as a key player in pyroptosis. This recently characterized class of pore-forming effector proteins is pivotal in orchestrating processes such as membrane permeabilization, pyroptosis, and the follow-up inflammatory response, which are crucial self-defense mechanisms against irritants and infections. GSDMs have been implicated in a range of diseases including, but not limited to, sepsis, viral infections, and cancer, either through involvement in pyroptosis or independently of this process. The regulation of GSDM-mediated pyroptosis is gaining recognition as a promising therapeutic strategy for the treatment of various diseases. Current strategies for inhibiting GSDMD primarily involve binding to GSDMD, blocking GSDMD cleavage or inhibiting GSDMD-N-terminal (NT) oligomerization, albeit with some off-target effects. In this review, we delve into the cutting-edge understanding of the interplay between GSDMs and pyroptosis, elucidate the activation mechanisms of GSDMs, explore their associations with a range of diseases, and discuss recent advancements and potential strategies for developing GSDMD inhibitors.

## Introduction

The recently identified gasdermin (GSDM) protein family is pivotal in the modulation of pyroptosis, a specialized form of programmed cell death (PCD). In humans, six paralogous genes have been identified: GSDMA-E, and DFNB59 (Table 1).^1,2^ The function of GSDMs in pyroptosis is well-established, and GSDMA-E have been shown to undergo proteolytic processing, resulting in the release of N-terminal (NT) fragments that assembles into pores at the plasma membrane (PM).^3–6^ These GSDM pores possess the ability to perforate both PM and mitochondrial membranes, triggering inflammatory cell death. Additionally, they facilitate the extracellular secretion of cellular elements such as inflammatory cytokines^7^ and mitochondrial DNA (mtDNA),^8^ which are known to participate in the pathogenesis of numerous diseases. Among the GSDMs, GSDMD has been the subject of extensive research and was initially recognized as a pivotal mediator of inflammasome-triggered pyroptosis. Moreover, it is highly involved in multiple disease-associated inflammations. Upon activation of GSDMD, the linker region can be cleaved by caspase-1/11 (caspase-1/4/5 in human), allowing GSDMD-NT to separate from autoinhibitory structural domain, GSDMD-CT.^9^ GSDMD-NT forms transmembrane pores, releasing cytokines like interleukin (IL)-1β^10^ and IL-18,^11^ disrupting ion and water homeostasis,^12^ and thereby potentially exacerbating the progression of diverse inflammatory conditions.^5^

**Table 1:** The GSDM family: expression, functions, and implications for disease

GSDM family	Gene and chromosomal location	Aliases	Activating enzyme	Expression in cells/tissues	Biological function	Associated diseases	Refs
GSDMA	Human: GSDMA (17q21.1) Mouse: Gsdma1–3 (11D)	GSDM1, FKSG9	SpeB caspase-1 (non-mammals)	Esophagus, prostate, bladder, skin, gastric epithelium, CD4 T	Tumor suppresser; pyroptosis	Systemic sclerosis, IBD, asthma, alopecia	^2,19,145,147,209^
GSDMB	Human: GSDMB (17q21.1) Mouse: None	GSDML, PP4052, or PRO2521	Caspase-1, granzyme A	Digestive system, reproductive system, respiratory system, skin, bladder, spleen, NK cells, CD4 T, CD8 T	Tumor suppresser; pyroptosis	Breast cancer, asthma, IBD	^24,36,151,152,155,165–167^
GSDMC	Human: GSDMC (8q24.21) Mouse: Gsdmc1-4 (15D1)	MLZE	Caspase-8	Esophagus, vagina, skin, spleen, trachea, small intestine, colon	Pyroptosis	Metastatic melanoma	^17,35,169,176,529^
GSDMD	Human: GSDMD (8q24.3) Mouse: Gsdmd (15D3-E1)	GSDMDC1, DFNA5L, or FKSG10	Caspase-1/4/5/11, caspase-8, cathepsin G, neutrophil elastase	The vast majority of human organs and tissues, different types of leukocytes and T cells	Pyroptosis, NETosis, cytokines release	Sepsis, AD, AS, ARDS, IBD, EAE, FMF, HCC	^28,29,42,43,175,176,180,226,258,502^
GSDME	Human: GSDME (7p15.3) Mouse: Gsdme (6B2.3)	ICERE-1, DFNA5	granzyme B Caspase-1/3/7 (teleosts)	Small intestine, cochlea, placenta, heart, brain, kidney	Pyroptosis, anti-tumor immunity, cytokines release	Deafness, cancer	^16,33,34,47,183–186^
DFNB59	Human: DFNB59 (2q31.2) Mouse: Dfnb59 (2C3)	PJVK, GSDMF	Not known	inner ear, liver, intestine, lung, kidney, brain, testis, CD4 T, CD8 T	Not known	Deafness	^20,21,202,203^

*AD* Alzheimer’s disease, *ARDS* acute respiratory distress syndrome, *AS* atherosclerosis, *EAE* experimental autoimmune encephalomyelitis, *FMF* familial Mediterranean fever, *HCC* hepatocellular carcinoma, *IBD* inflammatory bowel disease

GSDMs are emerging as attractive checkpoints for immune response, inflammation, cancer, and autoimmune disorders, in addition to their involvement in a multitude of systemic conditions.^3,13,14^ In recent years, significant strides have been taken in the development of small molecule inhibitors targeting GSDMD. Several GSDMD inhibitors alleviated pathology in preclinical disease models.^5^ The encouraging results have accelerated the pace of developing GSDMD inhibitors, progressing from preclinical studies to human trials. Consequently, it is both crucial and opportune to examine the functions and mechanisms of novel GSDMs in a spectrum of illnesses and their potential clinical applications. Understanding which GSDMD inhibitors should be prioritized in trials for specific disease indications is becoming particularly urgent.

In the present review, we compile the interplay between GSDMs and pyroptosis, delineate the pyroptosis-independent functions of GSDMs, elucidate the mechanism underlying pore formation by GSDMs, and explore their significance in human health and the pathogenesis of diseases. We also discuss the disease areas where GSDMD inhibitors can be preferentially applied and the advantages and disadvantages of inhibiting GSDMD-mediated pyroptosis.

### Research history and milestone events in gasdermins

**Fig. 1:**
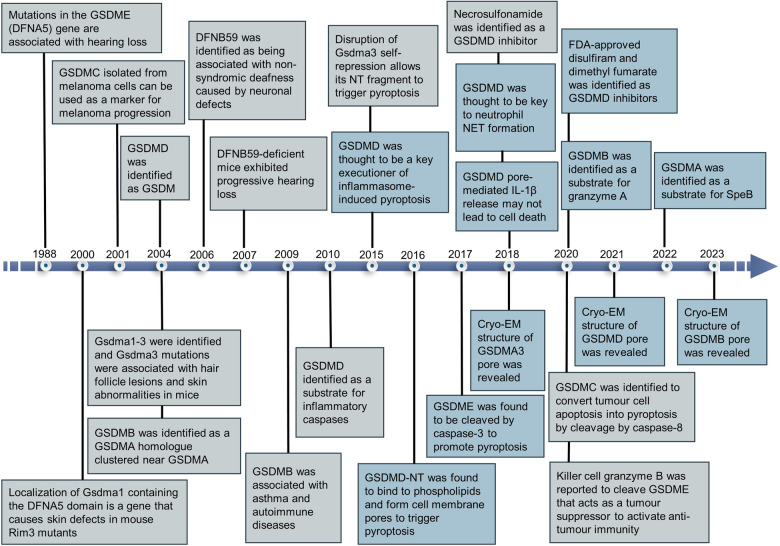
Highlights in the evolution of gasdermins development. Blue boxes denote seminal breakthroughs in gasdermin research

GSDMs represent a gene family with a conserved structural motif. Initial insights into GSDMs emerged in the early 2000s (Fig. 1).^15^ Saeki and colleagues cloned the mouse gene, GSDM, which bore the signature of the deafness autosomal dominant non-syndromic sensorineural 5 (DFNA5) gene.^15^ The term GSDM is derived from its selective expression in the mouse gastrointestinal tract and epithelial layers of the skin, an essential step in pinpointing the gene responsible for the Rim3 mutation in mice. The NT region of GSDM exhibited robust sequence similarity to DFNA5. In 1998, Laver et al. revealed an association between DFNA5 gene mutation and non-syndromic hearing loss.^16^ Following this discovery, the GSDM family expanded to include additional members, alongside proteins exhibiting GSDM-like characteristics.

In 2001, researchers isolated GSDMC (also known as MLZE) for the first time from mouse melanoma cells and observed that as the metastatic ability of the tumor increased, the expression of GSDMC rose accordingly,^17^ a finding that provided insights into the genetic variation underlying the progression of melanoma. Subsequently, in 2004, researchers discovered the Gsdma1–3 genes^18^ and noted that mutations in Gsdma3 were strongly linked to hair follicle disease and cutaneous anomalies in mice.^19^ In the same year, GSDMB was identified as a neighboring homolog of GSDMA, while GSDMD was characterized as part of the GSDM family.^18^ By 2006, the DFNB59 gene was found to be associated with hereditary deafness, and mutations in it may lead to non-syndromic hearing loss.^20^ In animal models, mice lacking DFNB59 developed progressive hearing loss,^21^ and analyses of the human genome similarly suggested that mutations in DFNB59 may cause non-syndromic deafness in humans.^22,23^ In 2009, two genomic studies revealed a link between variants within GSDMB and susceptibility to asthma and autoimmune disorders.^24,25^ Following this, Agard et al. in 2010 further elucidated the substrates of inflammatory caspases, stating that GSDMD is the most efficient and specific substrate for caspases under inflammatory conditions.^26^ The terminology of “pyroptosis” was first posited in 2001 to denominate a distinct mode of PCD that is reliant on inflammatory caspase-1. This process is distinctively typified by the induction of pore formation in the cell membrane, subsequent rupture, cellular swelling, and the dispersal of intracellular contents. The contents released during pyroptotic cell demise potentiated the inflammatory response and orchestrated an immune system activation.^27^ However, the role of GSDMs in pyroptosis remained unresolved.

It was not until 2015 that the relationship between GSDMs, inflammation, and cell death began to become clearer. Three independent studies uncovered the pore-forming capacity of GSDMD, portraying it as a major executor of pyroptosis and fostering inflammatory responses.^7,28,29^ Moreover, Shi et al. further demonstrated that other proteins within the GSDM family also possess pyroptosis-inducing activity in their conserved NT domains.^28^ For example, gain-of-function mutation in Gsdma3 lifts self-repression, enabling the NT domain to activate pyroptosis. Subsequently, several investigations in 2016 further revealed that the NT domains of GSDMD are able to create pores by forming oligomers in PM, thereby initiating the process of pyroptosis.^9,30–32^ As research progressed, GSDME,^33,34^ GSDMC,^35^ GSDMB,^36–38^ and GSDMA^39,40^ were also found to mediate cell pyroptosis. In 2017, Wang et al. uncovered a novel function for GSDME in the process of pyroptosis. They found that GSDME could transform the caspase-3-mediated apoptotic pathway triggered by tumor necrosis factor (TNF) or chemotherapy agents into a pyroptotic pathway.^33^ Caspase-3 was able to specifically target GSDME for cleavage by cleaving Asp270 in the linker, generating GSDME-NT that forms pores in PM, which triggers pyroptosis.^33,34^ To delve into the mechanisms underlying GSDM pore formation, Ruan and colleagues conducted cryo-electron microscopy (cryo-EM) analyses of mouse GSDMA3-NT pores in 2018.^41^ The GSDMA3 pore has a 27-fold symmetry and is structured as an intact antiparallel β-barrel consisting of 108 strands of β-strands. Charles L. Evavold et al. discovered that GSDMD can independently facilitate the release of IL-1β without causing cell lysis, implying the presence of a repair mechanism specific to the GSDMD pore. Judy Liberman and Hao Wu’s team hypothesized that the double-ring pore structure formed by GSDMA3 may be associated with pore repair.^10^ In the same year, the findings of Sollberger and colleagues, as well as Chen and team revealed the function of GSDMD in regulating neutrophil extracellular trap (NET) formation,^42,43^ which led to the realization that GSDMD appears to be a more sophisticated modulator of the inflammatory process than had been anticipated. Following this, researchers identified inhibitors of GSDMD, such as necrosulfonamide (NSA),^44^ as well as existing FDA-approved drugs such as disulfiram (DSF)^45^ and dimethyl fumarate (DMF).^46^ The therapeutic efficacy of these compounds in the context of inflammatory disorders has been persuasively demonstrated. In 2020, research highlighted the role of GSDME as a tumor suppressor, which augments anti-tumor immunity through the induction of pyroptosis.^47^ The role of GSDMC in tumors was also reported. GSDMC was specifically cleaved by caspase-8 to produce GSDMC-NT, which formed pores in PM and converted apoptosis to pyroptosis.^35^ Meanwhile, GSDME^47^ and GSDMB^36^ were identified as substrates for granzyme B and granzyme A, respectively. As of 2022, researchers have revealed the mechanism of GSDMA activation.^39,40^ It was shown that the SpeB protease of staphylococcal group A (GAS) could cleave at the Gln246 site of GSDMA, releasing the NT domains, which in turn initiate pyroptosis. Beyond their involvement in pyroptosis, GSDMs are also integral to the preservation of tissue homeostasis. For example, in the context of inflammatory bowel disease (IBD), Rana et al. found that GSDMB regulates the phosphorylation of local adhesion kinases, thereby contributing to epithelial maintenance and damage repair.^48^ In addition, Zhang et al. reported that GSDMD promoted mucin secretion from goblet cells in the colon, which was essential for maintaining intestinal mucosal homeostasis.^49^ In 2023, Zhong et al. determined the cryo-EM structure of the 27-fold-symmetric GSDMB pore, revealing that its internal and external pore diameters are ~160 and 270 Å, respectively.^38^ The GSDMB pore, reminiscent of the architectures observed in GSDMA3^41^ and GSDMD,^50^ is composed of a coronal ring in addition to a transmembrane β-barrel ring. It is worth pointing out that the cleavage products of GSDMs do not always result in cell death. Specifically, NT domains of GSDMB isoforms 3 and 4 are able to cause pyroptosis, whereas isoforms 1, 2, and 5 are not,^37^ suggesting that cells may inhibit and evade pyroptosis by generating noncytotoxic isoforms of GSDMB. Ongoing studies of the GSDM family have delved into the mechanisms of PCD and inflammation, highlighting the necessity for comprehensive inquiries into the roles and operational mechanisms of these molecules in both health and disease states.

### Mechanisms linking the versatile gasdermins in pyroptosis

Recent investigations have elucidated a pivotal role for GSDMs in managing the intricacies of cell death orchestration, in particular, their remarkable property of regulating pyroptosis through the formation of GSDM pores. It is currently known that in addition to DFNB59, the remaining proteins share similar structures, featuring an NT pore-forming domain and a CT regulatory domain. The NT domains of GSDMA-E are able to penetrate the lipid bilayers and form pores,^28,29,33,35,36,39,40^ whereas DNFB59 no longer possesses this pore-forming ability, yet it remains responsive to inflammatory and infectious triggers, retaining its activity.^51^ Moreover, the formation of GSDM pores is intricately linked to a suite of processes, including NETosis, autophagy, necroptosis, and apoptosis. GSDMD cleaved by caspase-11 or neutrophil elastase (NE) is involved in neutrophil NETosis,^42,43,52,53^ while cathepsin G can also cleave GSDMD, albeit without triggering cell death, instead fostering neutrophil inflammatory responses.^53^ The activation of GSDMA,^54^ GSDMD,^55,56^ and GSDME^57^ regulates mitochondrial oxidative stress, elucidating their participation in mitophagy. Furthermore, the processing of GSDME by caspase-3 gives rise to the initiation of secondary necrosis in cells undergoing apoptosis.^34^ The NT domains of GSDMD initiates the liberation of mitochondrial reactive oxygen species (mtROS), triggering pyroptosis through the NLRP3/GSDMD axis or necroptosis along the mixed-lineage kinase domain-like pseudokinase (MLKL) pathway.^58^ Additionally, NT domains of GSDMD and GSDME direct targeting to mitochondria, which aids in the facilitation of the release of cytochrome c, thereby activating caspase-3-mediated apoptosis.^59^

### Overview of pyroptosis

Pyroptosis represents a newly discovered PCD that is critically dependent on PM pores formed by the GSDM family, frequently, although not invariably, following the activation of inflammatory caspases.^60,61^ The development history of pyroptosis is described in detail by ref. ^6^ Pyroptosis manifests as a sustained cellular expansion that ultimately culminates in membrane rupture, thereby releasing intracellular contents and eliciting robust inflammatory responses, and is involved in many pathophysiological processes. Specific inflammasomes and inflammatory caspases are triggered by different signals, and caspases execute their function by excising the connecting segment of GSDMs, which disengages the NT and CT domains. This dissociation allows for the modulation of the pore-forming activity of the NT domain that is suppressed by the CT domain at a steady state. In response, the lipophilic NT domain undergoes translocation to the PM, where it associates with acidic phospholipids, like phosphoinositides, within the cytosolic leaflets of PM. This interaction promotes oligomerization, culminating in the assembly of ring-shaped pores. Such GSDM-mediated pores allow the release of cellular contents and cause cell lysis as the pores continue to accumulate. Initially, the researchers reported two pyroptosis pathways: the canonical pathway, which is caspase-1 dependent, and the non-canonical pathway, activated through caspase-4/5/11. With the continued study of GSDMs, two additional pyroptosis pathways, involving the apoptotic caspases and the granzymes, were revealed. Here we focus on the first two pathways, and the latter two can be found in the “Gasdermins and pyroptosis” section.

#### Canonical pathway

**Fig. 2:**
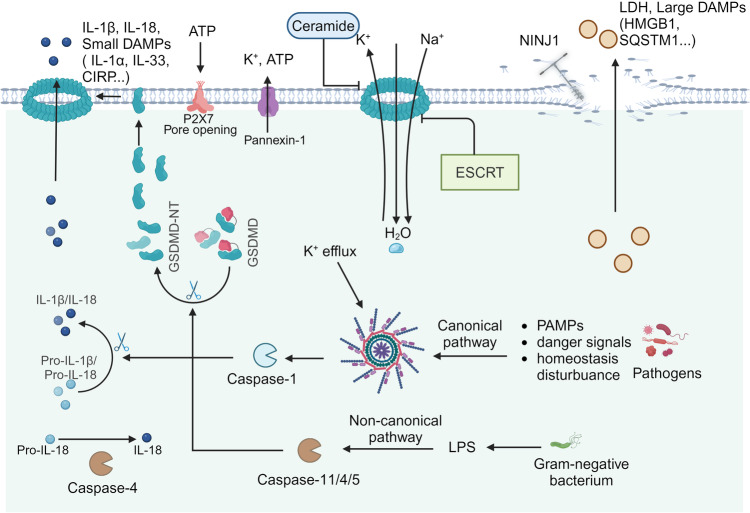
GSDMD-mediated pyroptosis and rupture of the plasma membrane. DAMPs and PAMPs stimulate inflammasome assemblies formation and caspase-1 activation in the canonical pyroptosis pathway, as well as caspase-11 activation (human caspase-4/5) in the non-canonical pyroptosis pathway. Upon activation, caspase-1/11/4/5 cleaves GSDMD to produce GSDMD-NT. Simultaneously, caspase-1 matures pro-IL-1β and pro-IL-18. Moreover, caspase-4 non-canonical inflammasome also matures IL-18. GSDMD-NT assembles into oligomeric pores on the plasma membrane, mediating the release of small molecules such as IL-1β/IL-18, and K^+^ efflux facilitates NLRP3 inflammasome assembly. Water permeates pyroptotic cells, leading to swelling and NINJ1-dependent PMR. Concurrently, the discharge of large intracellular molecules, including LDH and DAMPs such as HMGB1, is observed. Moreover, the activation of caspase-11 results in the cleavage of Pannexin-1, which in turn facilitates ATP release and orchestrates P2X7-associated cell death. Two mechanisms can repair membrane damage induced by the GSDMD pore: endosomal sorting complexes required for transport (ESCRT); and ceramide for endocytic repair of the GSDMD pores

**Fig. 3:**
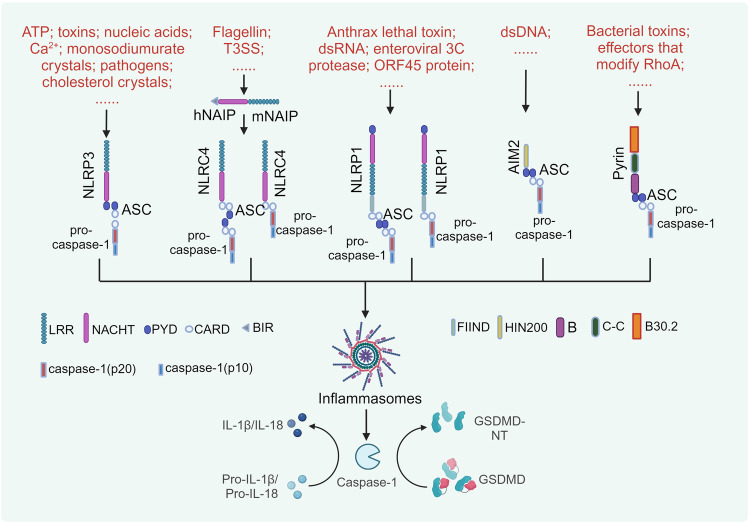
The activation mechanisms of canonical inflammasomes. The convergence of canonical inflammasome components is a crucial step in triggering pyroptosis. A diverse array of PAMPs and DAMPs, such as toxins and nucleic acids, serves as a trigger for NLRP3 inflammasome priming, leading to the recruitment of ASC and pro-caspase-1. The T3SS initiates NLRC4 inflammasome activation via NAIP, and NLRC4 mediates NLRP3 inflammasome activation by engaging ASC or through direct CARD-CARD domain interactions, which promotes the recruitment of pro-caspase-1 into the assembly complex. Anthrax lethal toxin stimulates the NLRP1 inflammasome, resulting in the activation of pro-caspase-1, which can occur either contingently or independently of ASC recruitment. The AIM2 inflammasome assembles upon detection of dsDNA from host or pathogenic sources. Pyrin inflammasome activation occurs due to bacterial toxins and RhoA-modifying proteins. Both AIM2 and Pyrin engage ASC-mediated signaling to orchestrate the activation of pro-caspase-1. Upon activation, caspase-1 executes its function by cleaving GSDMD and pro-IL-1β/pro-IL-18, thereby facilitating the release of these cytokines via a channel formed by GSDMD-NT

The canonical pyroptosis pathway is triggered by the assembly of the inflammasome, which activates caspase-1. This activation is then propagated through the cleavage of GSDMD, culminating in the release of IL-1β and IL-18 and triggering various physiological responses (Fig. 2).^6,62–65^ Inflammasomes represent multiprotein complexes activated to protect host cells from certain pathogens and endogenous danger signals (Fig. 3).^66–69^ The assembly of canonical inflammasomes begins with cytosolic pattern-recognition receptors (PRRs) that recognize pathogen-associated and damage-associated molecular patterns (PAMPs and DAMPs).^70^ Activated PRRs promote downstream type I interferon (IFN) production and pro-inflammatory cytokines release.^71–74^ Upon activation of host cells by bacteria or viruses, and so forth, PRRs such as nod-like receptor (NLR) family pyrin domain containing 3 (NLRP3), NLR family caspase activation and recruitment structural domain (CARD) containing 4 (NLRC4), NLR family pyrin structural domain containing 1 (NLRP1), Absent in melanoma 2 (AIM2), and pyrin, associate with pro-caspase-1 and adapter protein apoptosis-associated speck-like protein containing a CARD (ASC) to establish the canonical inflammasomes.^70,73–77^ Subsequently, mature caspase-1 is produced, distinct from the non-canonical pathway.^28,78^ Upon activation, caspase-1 performs a proteolytic conversion of pro-IL-1β and pro-IL-18 to their mature forms, IL-1β and IL-18, respectively. Complete GSDMD is also rapidly cleaved into two parts, GSDMD-NT and GSDMD-CT, in order to relieve the inhibitory constraint exerted by GSDMD-CT upon GSDMD-NT.^7,28,29^ GSDMD-NT promotes oligomerization in PM to form pores, triggering cell swelling and subsequent membrane rupture, which exposes the cellular contents and intensifies the inflammatory response.^3,30,79,80^

Exposure of inflammasomes to diverse stimuli initiates pyroptosis (Fig. 3). Distinct from other canonical inflammasomes, NLRP3 does not seem to directly identify a particular PAMP or DAMP. Extracellular adenosine triphosphate (ATP),^74,81^ pore-forming toxins (such as nigericin and maitotoxin),^82,83^ certain exogenous and endogenous particles,^84–86^ pathogen-associated RNA,^87–89^ bacterial and fungal toxins and components,^90,91^ intracellular Ca^2+^,^92–94^ and endoplasmic reticulum (ER) stress^95,96^ can trigger NLRP3, resulting in a decrease in the intracellular concentration of K^+^ and the efflux of cytosolic lysates from lysosomes, which in turn trigger mitochondrial dysfunction. The upstream immunosensor proteins for NLRC4 inflammasome activation are NLR family apoptosis inhibitory proteins (NAIPs).^97^ Investigations to date have revealed that the mouse genome harbors seven distinct NAIPs, whereas the human genome exhibits a singular NAIP gene.^98,99^ NLRC4 induces pyroptosis independently, dispensing with the requirement for the adapter protein ASC. Nonetheless, the engagement of ASC markedly enhances the propensity for NLRC4-induced pyroptosis.^100,101^ Bacterial flagellin,^102^ *S. Typhimurium* PrgJ, and *B. pseudomallei* BsaK^103^ can trigger NLRC4 activation, which are constituent proteins within the type III secretion system (T3SS). The precise mechanism underpinning NLRP1 activation remains elusive, with insights primarily derived from mouse studies.^6,104^ In contrast to humans that have only one NLRP1 gene, mice express multiple NLRP1 alleles, with NLRP1B being the main subject of study.^104^ NLRP1 is capable of being triggered by anthrax lethal toxin (LeTx),^105,106^ dsRNA,^107^ enteroviral 3C protease,^108^ ORF45 protein,^109^ dsDNA mimetic poly (dA:dT),^110^ ultraviolet B (UVB) radiation and the toxin-induced ribotoxic stress response (RSR).^111^ Interestingly, the initiation of NLRP1 is highly reliant on the activity of the proteasome, implicating proteolytic degradation as a pivotal process in NLRP1 activation.^106,112,113^ This is because NLRP1 cleavage releases the carboxyl-terminal effector domain from the inhibition of the amino-terminal effector domain, thereby triggering the formation of ASC specks and the activation of caspase-1.^114–117^ Self-cleavage of NLRP1 is an indispensable, albeit not standalone, requirement for the activation of NLRP1. The AIM2 inflammasome represents a distinct cytosolic innate immune sensor, variance from the NLR inflammasome, with its activation being predominantly mediated through the HIN200 domain, in response to damaged DNA, endogenous DNA aberrantly present within the cytosolic compartment, and exogenous DNA accumulated in the cytoplasm by intracellular pathogens.^118–121^ AIM2 does not contain a CARD domain, and thus its activation requires the assistance of ASC.^118^ Mefv-encoded pyrin functions as a phagocytic inflammasome sensor, responsive to the activation by bacterial toxins that manipulate RhoA. Like AIM2, pyrin plays a pivotal role in triggering inflammasome assembly through binding of the pyrin domain (PYD) to ASC.^122–124^ Furthermore, beyond the NLRP1 inflammasome, other complexes can participate in the canonical pyroptosis pathway, but do not independently mediate this process. The latest study has identified a pivotal function of NLRP11 in canonical pyroptosis of human macrophages.^125^ Gangopadhyay et al. found that NLRP11 engaged in a cooperative assembly of the NLRP3 inflammasome, with the absence of NLRP11 hindering the initiation of pyroptosis. Notably, the expression of NLRP11 is exclusive to humans, highlighting the distinctive intricacies of human inflammasome regulation.

#### Non-canonical pathway

Lipopolysaccharide (LPS), a prototypical PAMP, serves as an effective mediator in the progression of sepsis, which continues to be the principal cause of mortality. Intracellular LPS induces the activation of caspase-11/4/5 by directly binding to the NT CARD of these caspases (Fig. 2).^126–130^ Upon activation, caspase-11/4/5 cleaves GSDMD, resulting in the production of the GSDMD-NT, thereby forming pores in PM and directly promoting pyroptosis.^127,128^ Notably, this process can be activated secondarily by the release of IL-1β and IL-18 from NLRP3-mediated pyroptosis, termed non-canonical NLRP3 inflammasome activation.^29,131–133^ Initially, caspase-11/4/5 lacked the capability to cleave the precursor forms of IL-1β/IL-18^127,128^; however, recently, Shi et al. demonstrated that caspase-4/5 activated by LPS were capable of cleaving pro-IL-18 at a tetrapeptide cleavage site that coincides with the target site of caspase-1.^134^ The presence of a cytosolic LPS-specific PPR has emerged only recently. In human macrophages, the process of LPS-activated caspase-4 is dependent on NLRP11, an adapter protein that binds to LPS and caspase-4, thereby facilitating the assembly of a multiprotein complex.^135^ In addition, Furthermore, NLRP11 plays a role in NLRP3 inflammasome assembly,^125^ highlighting the intricate mechanisms by which human immune cells modulate the pyroptosis process.

Pannexin-1 stands as a pivotal protein in mediating macrophage death via a caspase-11-dependent non-canonical pathway.^136^ Cytosolic LPS triggers cleavage of pannexin-1 channels by caspase-11, leading to the subsequent release of ATP, which activates purinergic receptor P2X7 to facilitate the manifestation of cytolytic activity.^136^ This sequence of events leads to the efflux of intracellular K^+^, the activation of the NLRP3 inflammasome, and the secretion of IL-1β. Significantly, pannexin-1 modulates the canonical activation of the NLRP3 inflammasome independently of P2X7 via inducing K^+^ efflux.^6,136,137^ These observations imply that NLRP3 inflammasome could serve as a pivotal linkage between canonical and non-canonical pyroptosis pathways.

Beyond LPS, evidence exists for the activation of caspase-11 by various additional molecular triggers. Oxidized phospholipid 1-palmitoyl-2-arachidonoyl-sn-glycero-3-phosphorylcholine (oxPAPC) specifically promotes pro-inflammatory responses mediated by caspase-11 in dendritic cells (DCs).^138,139^ However, oxPAPC only triggers the release of IL-1β, leaving the cells in a hyperactivated state without cell death.^138,139^ The presence of lipophosphoglycan by *Leishmania* activates caspase-11 in macrophages, which in turn activates NLRP3 and caspase-1.^140^ Furthermore, secreted aspartyl proteinases (Sap)2 and Sap6 of *Candida albicans* were previously reported to also activate caspase-11,^141,142^ but current evidence indicates that this is mediated through the production of type I IFN which modulate caspase-11 expression, as opposed to a direct triggering of caspase-11 activation.^141,142^

### Gasdermins and pyroptosis

Members of the GSDM family display distinct patterns of tissue expression (Table 1). At different body sites, these members show differences in their respective abundance in sensing, recognizing, and defending against infections, especially in specific mucosal tissues.^4,15^ For example, GSDMA is active mainly in the skin, digestive and urinary systems; GSDMB is predominant in the skin, digestive and respiratory systems, and within immune cell populations; GSDMC exhibits mainly distribution across the skin, gastrointestinal tract, and vaginal epithelium; GSDMD demonstrates a broad distribution across most organs and immune cells; whereas GSDME is primarily localized to the central nervous system (CNS), placenta, heart, and small intestine. In addition, DFNB59 functions mainly in the inner ear and gastrointestinal tract. This variability in expression is closely related to the roles played by the GSDM family in various diseases. For example, GSDMB may be implicated in the pathogenesis of asthma,^143^ loss of DFNB59 function may lead to hearing loss,^16^ and widely expressed GSDMD is potentially implicated in the pathogenesis of various diseases across multiple organs and systems.^1,5,144^

#### Gasdermin A

GSDMA stands as the initial characterized member within the GSDM gene family, mapping to chromosome 17 at location 17q21.1. This gene is associated with defective skin and hair development in mice carrying the Rim3 mutation.^97,145^ Subsequent studies have revealed that mice with similar skin phenotypes possess three GSDMA homologs (Gsdma1–3), which preferentially specify expression within skin and epithelial tissues, including the epidermis, hair follicles, and gastric epithelium.^19,97,146,147^ The majority of mutant phenotypes in these mice are attributed to Gsdma3, with mutations in this gene causing intense skin inflammation and alopecia,^147^ and all of these mutations are localized to Gsdma3-CT, which exhibits gain-of-function mutations, revealing a role for functional NT domain in pyroptosis.^32^ Researchers elucidated the crystal structure of GSMDA3 pores in 2018, providing key insights into GSDM pore formation.^41^ GSDMA expression in humans is mainly restricted to esophageal, bladder, and skin epithelial cells, but is frequently extinguished in gastric cancer, implying that DNA methylation may contribute to the suppression of GSDMA transcription.^148^

**Fig. 4:**
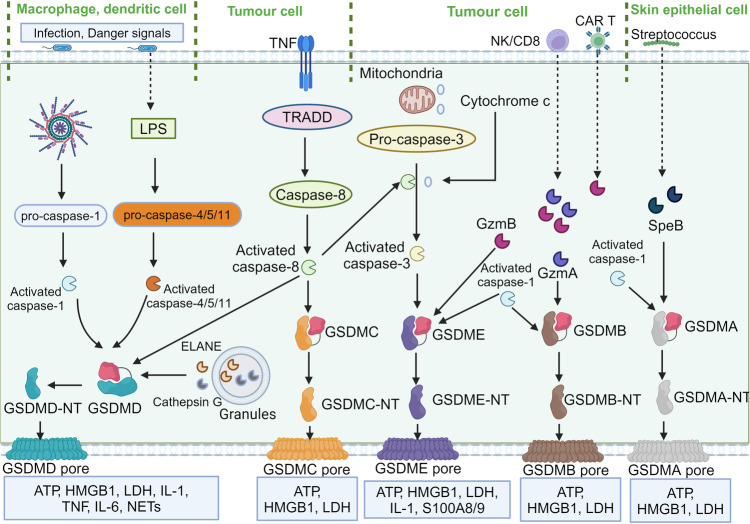
Molecular mechanisms of gasdermins activation. In response to microbial invasion, canonical inflammasomes and in response to LPS, non-canonical inflammasomes, respectively, trigger the activation of inflammatory caspases—caspase-1, -4, -5, and -11, resulting in GSDMD cleavage and generation of GSDMD-NT, followed by the formation of GSDMD pores. GSDMD is also processed by NE and cathepsin G released from neutrophil granules. *Yersinia* infection initiates caspase-8 to cleave GSDMD. Additionally, the degradation of GSDMC and GSDME is achieved through the action of caspase-8 and caspase-3, respectively, contributing to the transition from apoptotic to pyroptotic cell death. Caspase-8 and cytochrome c are involved in caspase-3 activation. Killer cells secret GzmA and GzmB, which directly cleave GSDMB and GSDME, respectively, to provoke pyroptosis. Secreted by group *A Streptococcus*, SpeB functions as a cysteine protease that specifically targets GSDMA, thereby initiating the pathological cascade leading to pyroptosis. Caspase-1 can also cleave GSDMB, GSDMA, and GSDME

GSDMA is associated with autophagy and pyroptosis. Mutations conferring gain-of-function lead to mitochondrial stress and increased ROS, and the NT domain exhibits pro-autophagic activity that induces an increase in LC3-II.^149^ Distinct from other GSDM family members, the proteases responsible for activating GSDMA-mediated pyroptosis have only recently been characterized, unveiling their important implications in host immune responses.^39,40^ Group A *streptococcus* (GAS), represents a pivotal skin pathogen responsible for a substantial burden of morbidity and mortality globally.^40^ Upon invasion by the GAS pathogen, SpeB undergoes autocatalytic cleavage to generate an active protease that directly proteolytically targets and cleaves GSDMA at the Gln246 site (Fig. 4),^39,40^ releasing an activated NT domain that promotes pyroptotic cell death in compromised cells. This process results in the initiation of local inflammatory responses and the subsequent eradication of pathogens, highlighting the critical role of GSDMA in host immunity. The absence of Gsdma1 or mutations/suppression of SpeB impedes the activation of GSDMA, triggering a localized immune response that propagates systemically, culminating in multi-organ infections. Investigation into the potential for GSDMA to exert a comparable function in diverse inflammatory disorders would be inquiries worth pursuing. Interestingly, in non-mammals, such as birds, amphibians, and reptiles, GSDMA undergoes cleavage by caspase-1. Consistent with the caspase-1-mediated cleavage of GSDMD in mammals, the tetrapeptide sequence within GSDMA is essential for its processing by caspase-1.^150^ This has led to a renewed understanding of the precision and complexity of the regulation of the immune system by GSDMs from an evolutionary perspective.

#### Gasdermin B

GSDMB, similar to GSDMA, also maps at 17q21.1, but GSDMB has not been identified in rodents.^143^ Compared to GSDMA, GSDMB exhibits a more extensive expression profile, primarily in airway and gastrointestinal epithelium, esophagus, stomach, liver, small intestine, and immune cells,^151–154^ but the question of whether these isoforms exhibit tissue- or cell-specific expression patterns remains unresolved. There is a significant correlation between polymorphisms in GSDMB and the propensity to develop chronic inflammatory disorders such as IBD, type I diabetes, and asthma.^24,151,155,156^

GSDMB enhances caspase-4 activity during non-canonical pyroptosis,^156^ indicating its potential role in inflammation. Previous evidence suggests that GSDMB could not be cleaved by inflammatory caspases but by apoptotic caspase-3/6/7, and the cleaved NT product may not contain an intact NT domain for pore formation or direct involvement in inflammation.^157^ In a study published recently, it has been demonstrated that during the process of apoptosis, activated caspase-7 is capable of cleaving the GSDMB protein at residue D91. The cleaved GSDMB fragment (92–417 aa) effectively inhibits the binding of the GSDMB fragment (1–91 aa) to caspase-4, thus preventing non-canonical pyroptosis.^158^ However, recent studies have presented a different view. In airway epithelial cells, GSDMB is susceptible to cleavage by inflammatory caspase-1, which liberates NT domains capable of triggering pyroptosis^159^ (Fig. 4). Granzyme A, exuded by cytotoxic T lymphocytes and natural killer cells, initiates the cleavage and activation of GSDMB, leading to pyroptotic cell death in tumor cells.^36^ This suggests additional evidence for its direct involvement in pyroptosis. However, Hansen et al. proposed a mechanism by which the enteropathogen *Shigella flexneri* secretes IpaH7.8, which is capable of ubiquitinating GSDMB and facilitating its degradation through the 26S proteasome pathway.^160^ Yin and colleagues delve into the interplay between IpaH7.8 and GSDMB, elucidating the molecular mechanisms underpinning GSDMB ubiquitination and its subsequent inhibition by IpaH7.8.^161^ This strategy counters the cytolytic effects of granzyme A on GSDMB to offer a protective buffer against bacterial elimination, and instead asserts a microbicidal role by targeting phospholipids on the bacterial plasma membrane. Remarkably, IpaH7.8 is also able to ubiquitinate the human GSDMD protein (but not the mouse) and direct its degradation via the proteasome pathway.^162^ This property may reveal why Shigella is able to trigger hemorrhagic gastroenteritis in primates but does not show similar symptoms in rodents. In recent times, the structure of the GSDMB-IpaH7.8 complex has been elucidated through the combined efforts of ref. ^163^ and ref. ^38^ employing cryo-EM and X-ray crystallography, respectively. This advance may provide insights into the questions posed above. The revealed structure features a complete GSDMB in an autoinhibited conformation, in conjunction with an IpaH7.8 leucine-rich repeat (LRR) domain that interacts with the GSDMB-NT. Notably, the IpaH7.8 LRR domain exhibits a specific recognition of an acidic motif within the α1 helix in the C terminus of GSDMB-NT, which includes residues E15, D17, and D21, acting as key structural determinants.

The GSDMB gene in humans gives rise to a family of at least six splice isoforms, each featuring a unique structural blueprint. Isoform 5 is characterized by the presence of the CT domain only, whereas isoforms 1-4 and 6 possess both the NT and CT domains that are conserved across the family. The linker sequences bridging these domains exhibit heterogeneity in length and amino acid composition among the various isoforms. Isoforms 4 and 6, by integrating a consensus sequence derived from exon 6 into their interdomain linkers, demonstrate strong pyroptosis-inducing capabilities. Conversely, isoforms 1, 2, and 3 lack the ability to trigger cell death.^163^ However, two other studies point out that GSDMB3, like GSDMB4, also has pro-pyroptosis activity, as GSDMB3 also has a stable band motif encoded by exon 6.^37,164^ These research discrepancies warrant further investigation. However, these findings hold significant value in elucidating the intricate roles of GSDMB isoforms in disease pathogenesis and in informing the future design of targeted GSDMB therapies.

Moreover, increased expression of GSDMB is observed in multiple cancers, spanning cervical, breast, gastrointestinal, and liver cancers, and its high expression is linked to an adverse prognosis,^165–167^ which may be related to the nuclear translocation and transcriptional regulatory functions of GSDMB,^151^ which may function independently of its role in pore induction. This phenomenon of acting independently of pyroptosis has also been validated in IBD. Epithelial-derived GSDMB preferentially populates genetic pathways linked to cell proliferation, migration, and adhesion, rather than pyroptosis, and can promote epithelial recovery and mucosal wound healing.^48^ The differential engagement of GSDMB in intestinal epithelial cells (IECs), mediating both pyroptosis and pro-restitution, epitomizes the intricate functional repertoire of a solitary protein within a discrete cellular context. Elucidating the underlying mechanisms of this multifaceted activity presents a compelling target for further investigation.

#### Gasdermin C

GSDMC, mapping on chromosome 8 (8q24.21), is initially detected in metastatic mouse melanoma cells, functioning as a biomarker indicative of melanoma progression.^17,18^ There are four Gsdmc homologs (Gsdmc1-4) in the mouse genome.^2^ GSDMC is expressed within various tissues, including the trachea, small intestine, colon, esophagus, skin, spleen, and vagina.^13,14^ Downregulation of GSDMC has been shown to inhibit the proliferation of colorectal cancer cells, suggesting a potential role in gastrointestinal cancers.^168^ In contrast, a separate study revealed that GSDMC was repressed in esophageal squamous cell carcinomas, suggesting that it may play a tumor-suppressive role.^153^ Synthetic truncations of GSDMC-NT have been demonstrated to provoke pyroptosis,^32^ as well as intracellularly GSDMC is cleaved by caspase-8 to generate the GSDMC-NT fragment that induces pyroptosis (Fig. 4).^35,169^ Hou et al. have uncovered that in breast cancer cells, GSDMC has the capacity to transform apoptosis into pyroptosis, a process that is promotive for tumor necrosis.^35^ In an oxygen-deprived environment, the p-signal transducer and activator of transcription 3 (STAT3) interacts with PD-L1, culminating in its nuclear translocation and enhancement of GSDMC transcription. As GSDMC expression rose, TNF promotes cleavage of GSDMC by caspase-8, generating GSDMC-NT that facilitates pyroptosis. In addition, the cellular metabolite α-ketoglutarate (α-KG) orchestrates the assembly of the DR6 receptosome in tumor cells, creating a molecular platform that enables efficient proteolysis of GSDMC by activated caspase-8, thereby triggering pyroptosis.^169^ These findings not only deepen our comprehension of the mechanisms underlying cell death and provide possible potential novel therapeutic tactics for cancer therapeutics.

Moreover, GSDMC participates in type 2 immune responses, as demonstrated by the increased expression of Gsdmcs in an in vivo model of worm-elicited type 2 immunity. Moreover, the overexpression of Gsdmc2 in human embryonic kidney 293 (HEK293) cells triggers pyroptosis.^170^ This pyroptotic mechanism could potentially facilitate the release of antiparasitic factors by IECs, aiding in the elimination of worms. This is consistent with the role of the GSDM family as executors of pyroptosis. However, Zhao et al. offered a contrasting perspective, suggesting that although GSDMC gene expression was highly increased in IECs following worm infection, it primarily functioned through a pyroptosis-independent pathway.^171^ They proposed that STAT6 O-GlcNAcylation regulated membrane pore formation by GSDMC-NT in IECs, which promoted IL-33 unconventional secretion as an alarm response, thereby potentiating the development of type 2 immunity. Notably, this pore-forming but non-lytic feature is different from the non-pore-forming feature of GSDMB found by ref. ^48^ The proteases that cleave GSDMC and the mechanism prevents intestinal pyroptosis following the formation of GSDMC-NT pores remain to be determined.

#### Gasdermin D

GSDMD stands out as the most extensively studied member within the GSDM family, being located on chromosome 8 at region 8q24.3.^9,172^ This protein exhibits broad tissue and immune cell distribution.^153,173^ GSDMD comprises a 31 kD NT pore-forming domain and a 22 kD CT suppression domain.^9,172^ An interdomain linker harbors a cleavage site, which is identified as D276 in murine GSDMD and D275 in human GSDMD. Upon activation, this linker is severed, resulting in the dissociation of the GSDMD-NT from the GSDMD-CT.^77^ Upon release, GSDMD-NT is inserted into the PM and oligomerizes, leading to pore formation, cytokines release, and interference with ion and water regulation.^10–12,174^ The intact GSDMD protein exhibits an inactive state, which is a consequence of its CT domain interfering with the interaction with its NT domain. However, GSDMD-NT exhibits high toxicity towards bacteria, indicating a potential direct engagement with cell membranes and subsequent lysis.^32^ This prediction is corroborated by the observation that GSDMD-NT binds with high affinity and specificity to phosphoinositides and cardiolipin, as well as forms extensive pores.^9,30–32^ Most of the pores feature an inner diameter within the range of 10–14 nm and are composed of roughly 16 symmetrical protomers.^32^ In contrast to characterized pore-forming proteins, GSDMD is uniquely positioned to induce cell lysis starting from the intracellular compartment of mammalian cells, a property that is linked to the asymmetric distribution of phosphoinositides within the PM.^30,32^

GSDMD is subject to proteolytic cleavage and subsequent activation by different molecules (Fig. 4). Inflammasome-mediated activation of caspase-1 through diverse canonical pathways, and LPS-mediated caspase-11/4/5 activation result in intense cleavage of GSDMD.^63^ Under suitable conditions, activated caspase-8 also cleaves GSDMD, a process that can occur in *Yersinia spp*. infection,^175–177^ in which the activities of TGFβ-activated kinase 1 (TAK1) and IκB kinase (IKK) are blocked. Furthermore, GSDMD can be cleaved by caspase-3. In contrast to the caspases described above, activated caspase-3 targets GSDMD at its NT domain, thereby preventing the assembly of functional GSDMD-NT pores.^178^ Among these caspases, caspase-1 stands out as the most effective catalyst for GSDMD cleavage, with caspase-8 demonstrating the least impact, possibly functioning as a contingency mechanism when other caspases are compromised.^179^ Finally, beyond these caspases mentioned above, neutrophils engage in the cleavage and activation of GSDMD through neutrophil elastase (NE) and cathepsin G. Cytosolic protease inhibitors, such as Serpinb1a and Serpinb6a, usually modulate cathepsin G-mediated pro-inflammatory responses by exerting inhibitory control.^42,43,53,180^ Notably, mutations contribute to GSDMD activation, with alterations in three amino acids within the CT domain that interfaces with the NT domain (L292, Y376, and A380), leading to the autonomous activation of GSDMD and cell death in mice.^181,182^ The corresponding human amino acids (L290, Y373, and A377) yield similar findings.^32^

Progressive studies of GSDMD have revealed that its function is not limited to its association with the process of pyroptosis, but that it can also independently carry out various biological roles, such as promoting the unconventional release of cytokines and the formation of NETs. GSDMD has been linked to the development of numerous diseases, and several inhibitors targeting GSDMD have been characterized and shown therapeutic efficacy in disease models. These findings will be discussed in greater detail subsequently.

#### Gasdermin E

GSDME maps on chromosome 7 (7p15.3), and it is initially perceived to be associated with hereditary hearing loss, devoid of any involvement in inflammatory processes.^16,183–185^ GSDME mRNA is distributed within various tissues such as the cochlea, placenta, heart, brain, and kidney.^16,186^ As research advances, GSDME is identified as a regulator of both apoptosis and pyroptosis.^33,34,187,188^ This protein can be cleaved by caspase-3, with evidence suggesting it plays a role in the induction of secondary necrosis following apoptotic triggers.^34,187,188^ With the lack of GSDME, cells exhibit a propensity to fragment into minute apoptotic vesicles rather than undergoing complete lysis. Alternatively, GSDME can target the mitochondrial membrane, prompting the efflux of cytochrome c and contributing to the generation of apoptotic cells.^59^ The above occurs when GSDME is expressed at low levels. However, at high levels of GSDME expression, caspase-3 activation leads to the cleavage of the protein, resulting in cell membrane perforations, cell swelling, rupture, and death.^189^ One study reveals that in the absence or non-function of caspase-1, pyroptosis in cells can still be triggered, independent of GSDMD activation.^190^ It is possible that caspase-8 triggers this death by activating caspase-3, which subsequently cleaves GSDME.^190,191^ Furthermore, in neutrophils, serine protease PR3 released by granules can promote the processing of GSDME by cleaving caspase-3.^192^ More interestingly, GSDME can serve as a strategic node positioning upstream of caspase-3, bridging exogenous and endogenous apoptotic pathways. This positioning augments caspase-3 activation, establishing a self-amplifying positive feedback loop.^189^ Interestingly, in teleosts, GSDME is subject to cleavage by caspase-1/3/7,^193^ where caspase-1-GSDME-mediated pyroptosis is highly efficient, whereas caspase-3/7-GSDME is less efficient in shifting cell death from apoptosis to pyroptosis.

Beyond its cleavage by caspase-1/3/7, GSDME can be activated by granzyme B, a cytotoxic lymphocyte-derived protein that infiltrates tumor cells (Fig. 4).^47^ This activation results in GSDME cleavage and triggers pyroptosis within the tumor cells. Notably, given that mutations in GSDME can result in hearing loss, the majority of these mutations are associated with the loss of the inhibitory CT domain, potentially leading to the initiation of cell death.^33,194^ Mechanisms regulating GSDME transcription have been reported recently. For instance, Wei et al. found that oxidized low-density lipoprotein (ox-LDL) stimulates the expression of GSDME in macrophages, leading to pyroptosis. Under the context of atherosclerosis (AS), STAT3 binds to the GSDME promoter, potentiating GSDME transcription and subsequent enhancement of caspase-3 activity, as well as the cleavage of GSDME. Consequently, this promotes the conversion of macrophage apoptosis to pyroptosis.^195^ Moreover, Pan et al. found that the transcription factor specificity protein 1 (Sp1) is involved in promoting pyroptosis induced by GSDME. Sp1 directly interfaces with the GSDME promoter at the -36 to -28 region, thereby potentiating the transcription of the GSDME gene. The knockdown of Sp1 can reduce cell pyroptosis induced by chemotherapeutic drugs.^196^ GSDME also emerge as a potential tumor suppressor,^197^ serving as a transcriptional target of p53 that is frequently epigenetically silenced through methylation in various malignancies.^198–200^ The absence of GSDME has been shown to compromise the efficacy of certain chemotherapeutic agents.^33,201^

#### DFNB59

As previously mentioned, the majority of GSDMs display a consistent architectural pattern with the exception of DNFB59, which maps on chromosome 2 (2q31.2). This distant relative within the GSDM family exhibits a truncated and non-homologous CT domain, setting it apart from other GSDMs in terms of sequence homology.^20,21,202^ DNFB59 exhibits widespread expression, with transcripts detected in various organs, including the lung, kidney, brain, inner ear, liver, intestine, and testis.^20,203^ This protein serves as a peroxisome-associated protein, crucial for the augmented proliferation of peroxisomes under oxidative stress conditions in hair cells and auditory neurons.^203^ DNFB59 senses sound-induced ROS and activates autophagic mechanisms to degrade damaged peroxisomes.^202^ The precise nature of DNFB59 remains elusive; whether it acts as a pore-forming protein or is inherently active is yet to be conclusive, attributable to its truncated CT domain, which might insufficiently suppress pore formation. Further exploration deserves to determine whether DNFB59 induces pores in peroxisomal membranes and, subsequently, to investigate whether associating proteins modulate its function by either inhibition or activation.^204^

Since the uncovering of GSDMs as key executors of pyroptosis, numerous studies have reported evidence linking the activation of GSDMs to various pathological contexts, considering the multiple functions of pyroptosis under different diseases.^13,14,63,205–208^ Moreover, spontaneous mutations that trigger GSDM activation have been implicated in several disorders, including alopecia (GSDMA),^19,147,209–211^ asthma (GSDMB),^151,155,212–214^ and hearing loss (GSDME/DFNB59).^16,20,183–185,215–219^ Accumulating evidence indicates that GSDMs could potentially participate in the modulation of infection and cancer,^35,36,42–47,180,220,221^ suggesting an intricate relationship between GSDMs-orchestrated pyroptosis and their non-lytic processes^48,52,222–226^ in the etiology and progression of these conditions.

### Gasdermin pore formation

#### Structural auto-inhibition in the full-length GSDMs

The latest findings reveal that GSDM-NT binds to phospholipids and triggers pyroptosis, which is not the case for full-length GSDM or GSDM-CT.^30,32,227^ Increased expression of GSDMD-CT effectively inhibited GSDMD-NT-induced pyroptosis under LPS stimulation.^32^ The crystal structure of GSDMs elucidates the mechanism of structural auto-inhibition: the CT domain of the full-length GSDMs folds onto the NT domain, preventing lipid interaction and subsequent pore assembly. The crystallographic datasets for mouse GSDMA3, human and mouse GSDMD, and human GSDMB proteins reveal that full-length GSDMs employ a mechanism of auto-inhibition facilitated by the intimate association of the CT domain with the NT domain. This interaction involves the α1 helix and β1–β2 hairpin of the NT domain engaging in extensive electrostatic and hydrophobic interactions with the CT domain, effectively preventing the activation of the GSDMs.^38,41,50,227^ The aromatic amino acids of the β1–β2 hairpin, Phe49, and Trp50 (Phe48 and Trp49 in mGSDMA3) are embedded into the hydrophobic pocket of the CT domain.^227^ Notably, GSDMA3 features an auxiliary contact surface that arises from the insertion of the α4 helix within the NT domain into a separate hydrophobic pocket of the CT domain.^32,41^ Unlike the GSDMD, the NT domain of GSDMB exhibits an elongated β-sheet structure containing ten antiparallel aligned β-strands (β1–β10) and is structurally ordered. α1 helices and their subsequent loops with neighboring β1/β2 hairpins constitute the main interaction interface with the GSDMB-CT domain.^38,163^ Mutational interference with the NT-CT domain interaction results in constitutive self-activation of the intact protein, implying the presence of a preserved mechanism for structural auto-inhibition within the GSDM family.

GSDMs found in fungi and bacteria (bGSDM) follow a similar strategy, leading to structural auto-inhibition. The short CT domain in fungi GSDMs interacts directly with the α1 helix and β1–β2 hairpin in the NT domain, and removal of the CT domain by caspases or protein hydrolysis leads to cytotoxicity exerted by the NT domain.^228^ Sequence analysis revealed the presence of 50 bGSDM homologs, distinct from eukaryotic homologs. Although the large α-helical CT domain required for structural auto-inhibition is lacking in *Bradyrhizobium tropiciagri* and *Vitiosangium sp*., they contain a structurally similar molecular substitute. The bGSDM caspase system is commonly present in bacteria and archaea, where bGSDM is cleaved by caspase-like proteases. It is worth noting that the pore structures formed by bGSDM are diverse and different in size from those of mammals, which may be products of specific internal substance releases. This reveals the functional conservation of GSDM in all life forms, from prokaryotes to eukaryotes.

#### Mechanism underlying the formation of GSDM pores

Cleavage by proteases facilitates the release of GSDM-NT, which subsequently translocates to PM and assembles into oligomeric pores. Electron microscopy has elucidated that the pore formed by GSDMD-NT has an inner diameter of ~12–20 nm, exhibits a symmetric subunit structure of approximately 16. Furthermore, GSDMD-NT pores isolated from liposomes are characterized by a molecular weight of approximately 24 kD.^9,32^ The use of cryo-EM and high-resolution atomic force microscopy (AFM) techniques further validate these data, revealing that the GSDMD-NT pore exhibits an average diameter of 20 nm with symmetry between 15 and 45.^31,229^ The diameter of the GSDMD-NT pore is appropriately sized to permit the transit of larger molecules, including IL-1 family cytokines and galectins.^10,174,225,230–232^ Further cryo-EM reveals that the macropore structure formed by human GSDMD-NT consists mainly of 33 (ranging from 31 to 34) subunits. These 33-fold macropores exhibited a full antiparallel β-barrel structure, which modestly exceeds the dimensions of the previously described 26–28-fold GSDMA3 pores and 26–30-fold GSDMB pores.^38,41,50,163^ Inserted into the membrane, the GSDMD-NT pore, the GSDMA3-NT pore, and the GSDMB-NT pore have a similar structure, all consisting of a coronary ring in conjunction with a transmembrane β-barrel ring. The NT domain is constructed like a left hand, in which the cytosolic globular structural domain serves as the palm, the α1 helix resembles the thumb, and the four protruding β-strands from the dual β-hairpins penetrated into the membrane constitute the fingers.^38,41,50,163^ The aggregation process of GSDM pores is mainly achieved by the interaction between cytosolic globular structural domains and transmembrane regions via a complex interplay of hydrophobic associations, electrostatic interactions, and hydrogen bonds, especially the α1 helices, which form a helical belt structure with head-to-tail linkages to stabilize the entire pore structure. This oligomerization pattern is retained in all GSDMs containing pore structures, including GSDMD, GSDMA3, and GSDMB.

Recent findings have documented that both GSDMD-NT and GSDMA3-NT can form not only ring-shaped pores in lipid bilayers and liposomes, but also smaller arc-shaped and slit-shaped pores.^229,233^ These pores composed of as few as two GSDMD-NT molecules are capable of admitting the passage of water and ions, and these assemblies also grow and fuse together.^229,233,234^ Smaller oligomers have the potential to organize into arc-shaped pores, potentially serving as conduits for the transit of small-molecule proteins across the PM. These arc-shaped channels are susceptible to adopt narrower slit-shaped channels due to the tension exerted by the external lipid bilayers, or they may continue to expand and fuse, eventually forming complete ring-shaped channels. This suggests that GSDMD pores are dynamic and non-homogeneous in structure, and that pore formation may occur along different pathways in parallel.

Previous studies of pore-forming GSDMs have revealed two stable ring-like oligomers that are not inserted into the membrane, termed “prepore”, and found in both GSDMD and GSDMA3.^41,50,235–240^ Comparison of the structures of GSDMD prepore and pore highlights conservation in diameter, with the precursor being approximately 40 Å shorter in height. This discrepancy is proposed to arise from a conformational change involving the globular domains, which undergo a rigid-body rotation relative to the membrane as the pore is formed.^50^ The globular domain within the prepore presents an autoinhibited configuration, yet the organized transmembrane region aligns more closely with the conformation observed in the mature pore, suggesting a transition from the prepore to the pore.^50^ Thus, the presence of GSDMD prepore and GSDMA3 prepore implies that GSDMs may adopt a conserved and simple synergistic mechanism during membrane insertion. However, this unifying mechanism contradicts reports that GSDMDs form arc-like and slit-like assemblies on the membrane.^31,229^ These observations imply that GSDM-NT may independently insert into PM and subsequently oligomerize within PM, with additional GSDM-NT recruited subsequently to expand these pores. Employing experimental approaches, such as patch-clamp electrophysiology or direct observation of prepores in the membrane, will aid in elucidating the pore-forming mechanism of GSDMs further.

### GSDM-mediated cell rupture and membrane repair

GSDM-NT interacts with acidic lipids to generate pores; however, the exact mechanism driving this process is yet to be fully elucidated. Ruan et al. employed cryo-EM to illustrate the formation of single-ring pores of GSDMA3-NT at resolutions of 3.8 and 4.2 Å, respectively, as well as double-ring pores at 4.6 Å resolution.^41^ The structure of the GSDMA3 pore exhibits similarities to that of the GSDMD pore. The GSDMA3 pore is composed of 26–28 subunits, while the number of subunits in the GSDMD pore is approximately equivalent to that of GSDMA3 and GSDMB. The GSDMD pore exhibits an inner diameter of ~10–14 nm, a measurement that accommodates the release of IL-1β (4.5 nm), along with a subset of other small DAMPs, encompassing IL-18, IL-1α, IL-33, galectin-1/3, ATP, and the cold-inducible RNA binding protein (CIRP). The implications of varying pore architectures and dimensions in dictating the liberation dynamics of diverse cargoes are yet to be fully elucidated. However, it is estimated that assemblies comprising at least 10 GSDMD-NT subunits may suffice to facilitate the transit of IL-1β.^234^

Subsequent cellular rupture releases larger molecules, including LDH, DNA-binding histones, high mobility group box-1 (HMGB1), sequestosome 1 (SQSTM1), and perhaps even organelles, a process mediated by Ninjurin-1 (NINJ1) (Fig. 2).^241,242^ As an evolutionarily conserved cell surface protein, NINJ1 facilitates cell membrane rupture and the discharge of DAMPs.^243^ These DAMPs are detected by PRRs, which activates a cascade of immune responses, resulting in the attraction of immune cells and the triggering or augmentation of inflammatory reactions, which can ultimately promote the manifestation of inflammatory diseases. NINJ1 offers insights into the uncoupling of GSDMD-mediated cell death from plasma membrane rupture (PMR). In macrophages from both mouse and human, deletion of NINJ1 does not inhibit GSDMD pore formation, cell swelling, and death, yet PMR is impaired.^243,244^ The precise mechanism underlying which NINJ1 augments PMR remains undetermined; however, this function is contingent upon an amphipathic α-helix within the NT region of NINJ1 and the assembly of NINJ1 into oligomers.^243^ Moreover, the triggers for NINJ1-mediated PMR are inconclusive. Dondelinger et al. demonstrated that hypotonicity was sufficient to induce NINJ1 oligomerization and NINJ1-mediated PMR in mouse embryonic fibroblasts (MEFs), but this in vitro system may not fully mimic the true situation of PMR in vivo.^245^ Wang et al. hypothesized that the activation signal for NINJ1 might involve a form of membrane modification or ion channel activation rather than osmotic pressure disruption, because NINJ1-mediated PMR plays a global effect, but apoptotic cells do not undergo swelling in the current consensus.^246^ The phenotype linked to NINJ1 depletion shares similarities with those observed in cells subjected to pyroptosis inducers in an environment containing glycine,^10,174,247,248^ and one report indicated that glycine administration curtails the assembly of NINJ1 oligomers that are correlative with PMR.^244^ These results imply that NINJ1 could represent a crucial target through which glycine exerts its protective effects on cellular integrity.^244^

More recently, it has been observed that cells expressing GSDMD-NT do not always undergo cell lysis. Owing to the presence of repair mechanisms for PMR, GSDMD-NT pores do not consistently lead to pyroptosis and may merely release inflammatory cytokines without cell death.^10,174,249^ The influx of Ca^2+^ via GSDMD pores functions as a signaling mechanism for cellular initiation of PM repair, recruiting the endosomal sorting complexes required for transport (ESCRT) to remove pores from the PM, which are subsequently shed as ectosomes.^249^ PM repair by ESCRT-III allows for restricted pyroptosis while permitting limited GSDMD-dependent cytokines release. Recent research has uncovered a unique resistance mechanism against cell lysis, where Ca^2+^ influx prompts lysosomal exocytosis at the site of damage, releasing acid sphingomyelinase (ASM). Caspase-7 mitigates GSDMD pores and maintains cellular integrity by activating ASM, thereby generating substantial ceramide levels. These ceramides facilitate clathrin-independent endocytosis to internalize GSDMD pores and repair damaged membranes.^250^ Under these conditions, pores fail to trigger pyroptosis but rather facilitate the secretion of IL-1 through them, generating a state of cellular hyperactivation that correlates with an elevated capacity to prime adaptive immune responses.^138^ The mechanism of PM repair aligns with the function of living cells in releasing inflammatory cytokines, which also corresponds to the observation that hyperactivated cells exhibit fewer GSDMD pores compared to pyroptotic cells.^10^ Notably, other potential mechanisms exist to promote PM repair. Phospho-MLKL is eliminated from PM via flotillin-driven endocytosis or ALIX-syntenin-1 axis of exocytosis, thereby inhibiting necroptosis. Similar to ESCRT-III- and caspase-7-mediated PM repair,^251^ these mechanisms may ensure that only signals of sufficient strength lead to necroptosis, but whether they inhibit membrane damage caused by pyroptosis remains to be demonstrated.

### Gasdermins and mitochondrial damage

Mitochondria regulate cell death with their diverse metabolic functions and demonstrate an important role in pyroptosis.^252^ Beyond its established interaction with PM, GSDM-NT is also known to engage with membranes within the interior of the cell.^8,42,54,57,59,226,253,254^ The NT domains of GSDMD and GSDME are capable of targeting mitochondria, where they interfere with the integrity of both the inner and outer mitochondrial membranes and disrupting their functional roles. This interference results in the production of mtROS, the release of mtDNA, the dissipation of transmembrane potential, and the release of cytochrome c. These cumulative actions ultimately lead to the activation of caspase-3, facilitating the execution of both apoptosis and pyroptosis.^8,57,59^ Additionally, mtROS is instrumental in fostering RIPK1/RIPK3/MLKL-dependent necroptosis,^254^ while mtDNA promotes GSDMD pore formation,^8^ further facilitating pyroptosis. Moreover, mtDNA can be sensed by the AIM2 inflammasome, initiating pyroptotic cascades.^255^ Mitochondrial damage also activates the NLRP10 inflammasome, resulting in ASC specks formation and the release of cytokines, independent of mtDNA.^256^ These insights underscore the significance of mitochondrial dysfunction in orchestrating immune responses through enhancing pyroptosis, and emphasizes the indispensable roles of GSDMs as proximal executors in multiple pathways of cell death.

The GSDMD-NT demonstrates a marked preference for binding with mitochondrial and bacterial lipids, as well as cardiolipin, exhibiting a significantly stronger binding affinity compared to PM lipids.^30,32^ Upon activation of GSDMD, the onset of mitochondrial damage precedes damage to PM.^8^ Similarly, GSDMA-NT tends to accumulate preferentially within mitochondria, with a delayed and diminished presence at PM.^253^ This distinct subcellular distribution kinetics implies that GSDM-NT may initiate mitochondrial dysfunction prior to their penetration into PM.

## Novel pyroptosis-independent functions of GSDMs

A multitude of investigations have centered on the role of GSDMs in pyroptosis, but recently GSDMs have also been reported to act independently of this process. The assembly of GSDM pores is not invariably predictive of pyroptosis occurrence, and cells may survive after moderate GSDM pore formation due to PM repair mechanisms, weak inflammasome activation, and oxidized lipid stimulation.^50,249,257^ In the following, we present the novel pyroptosis-independent functions of GSDMs from three aspects: GSDMs in IL-1 release, GSDMs in NETosis, and GSDMs in non-immune cells.

### GSDMs in IL-1 release

The initial characterization of the functional linkage between pyroptosis and the release of IL-1β was established in macrophages,^10,174,258^ yet this view has recently been challenged. Evidence has mounted to suggest that in macrophages and neutrophils, IL-1β is discharged via pores formed by GSDMD rather than by pyroptosis, or that the secretion of IL-1β is GSDMD-independent.^259^ Notably, NINJ1 has been documented to coordinate the ionic and osmotic disruptions triggered by GSDMD pores, facilitating the terminal stages of PMR.^243^ Despite its pivotal role in PMR, NINJ1 is not required for the formation of GSDMD pores, corroborating the concept of GSDMD as a pivotal player in both pyroptosis induction and the unconventional secretion of IL-1β.

Neutrophils are considered to be complex cells with a range of important specialized functions that serve as first-line weapons in the innate immune system.^260^ They are the most abundant subtype of granulocytes and are capable of assembling diverse inflammasome platforms to release IL-1β to defense various microbial pathogens.^261,262^ Unlike macrophages, neutrophils are not susceptible to GSDMD-dependent pyroptotic lysis upon activation of inflammasomes to maintain their vitality for efficient microbial eradication, while still employing a GSDMD-dependent mechanism for the export of IL-1β.^42,263,264^ However, this was not evident until it is confirmed that GSDMD is a conduit for macrophage IL-1β secretion and pyroptosis. Before these findings, some researchers merely indicated that the engagement of specific inflammasome signaling pathways in neutrophils could result in substantial IL-1β secretion without pyroptosis, as assessed by LDH release.^137,263–265^ These studies prompt an investigation into GSDMD in neutrophils, ultimately demonstrating a critical function for this protein in the controlled secretion of IL-1β by myeloid leukocytes and revealed pyroptosis as a universally exhibited pro-inflammatory form of PCD.

The mature IL-1β cytokine is generated through the proteolytic cleavage of pro-IL-1β by caspase-1, representing the most conventional pathway for its production. However, neutrophils are rich in azurophilic granules, which opens up the possibility of an abundance of IL-1β precursor protein-cleaving enzymes. Additionally, pro-IL-1β may be cleaved by a spectrum of serine proteases (including NE, cathepsin G) stored in azurophilic granules, thereby yielding a biologically active form of IL-1β.^266^ The proficiency of neutrophils to produce biologically active IL-1β via the canonical pathway and other serine protease pathways underscores their significance as a prime generator of pro-inflammatory cytokines during diverse innate immune responses. As previously described, GSDMD, which can be cleaved by caspase-1/11/4/5, constitutes a component within various inflammasome signaling pathways. The GSDMD-NT pore, which accumulates on the plasma membrane, is a conduit for the direct release of IL-1β. Significantly, the caspase-4/11-induced aggregation of GSDMD-NT pores on the PM can facilitate K^+^ efflux, which is sufficient to prompt the secondary assembly and activation of NLRP3 inflammasomes, as well as the caspase-1-dependent cleavage of pro-IL-1β, culminating in the efflux of mature IL-1β through the GSDMD-NT pore.^28^ Recent electron microscopic and functional assessments of GSDMD-NT pore have revealed a mechanism that prevents pro-IL-1β release, with GSDMD-NT pore mediating mature IL-1β release through electrostatic filtration, thereby hindering pro-IL-1β fluxes.^50,225^

Nonetheless, an alternative viewpoint has been proposed, with Karmakar et al. reporting that GSDMD-NT was essential for the secretion of IL-1β by human and mouse neutrophils, but it does not migrate to the plasma membrane, nor does it augment membrane permeability or trigger pyroptosis.^226^ GSDMD-NT produced by activated caspase-1 is trafficked to azurophilic granules, resulting in the deployment of NE into the cytoplasm and the subsequent secondary GSDMD cleavage. These finding suggests that the abundance and compact arrangement of neutrophil granules may function as a diffusion obstacle, impeding the transport of GSDMD-NT to the inner leaflet of the PM. They demonstrated that neutrophils deploy IL-1β secretion through a mechanism that is contingent upon autophagy, based on the observation that neutrophils from autophagy-related 7 (ATG7)-deficient mice exhibited impaired IL-1β secretion.^226^ It is noteworthy to highlight that the IL-1 family can also be liberated through a pathway that is independent of GSDMD. Monteleone et al. discovered that the initial secretion of IL-1β from mouse neutrophils was facilitated by a mechanism dependent on GSDMD. However, subsequent releases of IL-1β in both in vitro and in vivo settings occurred independently of GSDMD.^267^ However, inflammasomes accelerate IL-1β release through caspase-1 and GSDMD activation. Many previous investigations have found that macrophages can secrete IL-1β via exosomes,^268,269^ secretory autophagy,^270^ or small extracellular vesicles,^271^ suggesting other novel pathways for IL-1β secretion without pyroptosis. Recently, Ratitong et al. have confirmed that neutrophils utilize exosome secretion as a conduit for the release of IL-1α cytokine,^272^ which suggests that extracellular vesicles such as exosomes are a critical mechanism for the secretion of IL-1 family by neutrophils.

It is noteworthy that IL-1α and IL-33 are able to be secreted in living cells via the GSDM pores.^224,232,273^ In human T cells, the GSDME-NT pores mediate the unconventional IL-1α release, with the NLRP3/caspase-8/caspase-3/GSDME axis pivotal in this process.^273^ GSDMD in airway epithelial cells and macrophages is susceptible to cleavage by allergenic proteases to generate a novel fragment, p40 GSDMD-NT (p35 GSDMD-NT in humans), which effectively promotes IL-33 release without accompanying cell death.^232^ In addition, GSDMs also promote the unconventional release of other inflammatory mediators, including ATP and HMGB1.^274,275^

### GSDMs in NETosis

In 2004, NETs, the nuclear chromatin complexes encompassing DNA, citrullinated histone H3, myeloperoxidase, NE, and cathepsin G, were uncovered and postulated to play a pivotal role in the innate immune response arsenal of neutrophils.^276^ The process of NETosis, characterized by the deployment of NET structures, marks a significant research front in the domain of neutrophil physiology,^277–279^ eliciting a robust cellular response that is currently a subject of intense scholarly examination.^280,281^ NETosis is a multifaceted biological pathway that entails the disruption of both nuclear and granular membranes, the decondensation of chromatin, and its amalgamation with granule components, culminating in the extrusion of condensed chromatin from neutrophils. The combined action of GSDMD and caspase-11 in LPS-induced NETosis drives nuclear membrane breakdown, chromatin relaxation, and rupture of the PM.^42,43^ GSDMD-NT interacts with azurophilic granules, the releasing granule proteins required for NETosis progression, where NE can further cleave GSDMD. The formation of GSDMD pores within the nuclear membrane permits the rupture of this barrier and the infiltration of caspase-11 into the chromatin, where caspase-11 mediates histone shearing and inactivation to enable DNA amplification. The collaboration of caspase-11 and GSDMD is indispensable for neutrophil PMR, undergone by neutrophils during the terminal stages of NET extrusion.^42,43^ Sollberger et al. concluded that NET formation did not require caspase-11 activation because the proteolytic activation of GSDMD was independent of caspase-11.^43^ They proposed that neutrophil serine proteases cleaved GSDMD, releasing activated and toxic NT domains. This finding is consistent with the report of Kambara et al.,^180^ wherein NE was demonstrated to cleave GSDMD, and together these two studies suggest that GSDMD has additional functions independent of inflammasomes.

In 2022, Chauhan et al. suggested that GSDMD might not be an indispensable factor for PMA-induced NETosis,^282^ contrary to the view of Sollberger et al. ^43^ The latter view was proposed in the context of LDC7559 inhibiting PMA-induced NETosis by targeting GSDMD, yet LDC7559 was subsequently demonstrated to inhibit PMA-induced NETosis not by directly targeting and inhibiting GSDMD, but rather by functioning as a potent agonist of the glycolytic enzyme phosphofructokinase-1 liver type (PFKL).^283^ In 2023, Stojkov et al. discovered that NET formation after C5a or LPS stimulation of mouse neutrophils was GSDMD-independent.^284^ Neutrophils from both wild-type (WT) and GSDMD^−/−^ mice exhibit equivalent kinetics and magnitude of response to NET-inducing agonists, a process that is independent of cell death. Furthermore, even under conditions of canonical inflammasome activation, which culminates in GSDMD cleavage and prompts NET production, the release of NETs does not necessitate the participation of caspases or GSDMD.^284^ The differences between these studies described above could be attributed to variations in the stimulation conditions, the timing of NET assembly measurement, and the methodologies utilized for quantifying cell death. In conclusion, these recent reports imply that the role of GSDMD in regulating NETosis is incidental rather than mandatory. Clearly, the relationship between GSDMD and NETs needs further scrutiny due to potential discrepancies in NETosis and NET formation.

### GSDMs in non-immune cells

GSDMD in immune cells has received extensive attention for mediating cell death and promoting inflammation that contributes to the manifestation of diverse diseases. Now the function of GSDMD on tissue homeostasis in non-immune cells is gradually being reported. Li et al. discovered that in osteoblasts, GSDMD was cleaved into non-lytic p20 products, a function that serves to forestall bone resorption and preserve bone homeostasis.^285^ At late stages of receptor activator of nuclear factor-κB (NF-κB) ligand (RANKL)-induced osteoclastogenesis, GSDMD undergoes cleavage to produce p20 products rather than the canonical p30, a process that is reliant on receptor-interacting protein kinase 1 (RIPK1) and caspase-8/3. The GSDMD p20 is selectively targeted to early endosomes, where it constrains the maturation of endolysosomes and inhibits bone resorption. This function is mediated by the protein’s propensity for oligomerization and its ability to regulate phosphoinositide turnover by combining with phosphatidylinositol 3-phosphate (PI(3)P). GSDMD^−/−^ mice and Gsdmd^fl/fl^ Lyz2^Cre+^ mice show osteoporosis, exhibiting significant reductions in trabecular bone volume and trabecular number. Zhang et al. reported that GSDMD was pivotal in the secretion of mucin and the establishment of the mucus layer within goblet cells.^49^ Specific deletion of GSDMD in IECs results in reduced mucus secretion accompanied by loss of the mucus layer, which undermines the integrity of the host-microbial interface and impairs the effectiveness of pathogen clearance from the mucosal surface. The mechanism is that stimulation of NLRP6 in goblet cells activates caspase-1/11, which in turn activates the GSDMD via a mechanism of ROS synthesis. GSDMD-NT facilitates mucin secretion via Ca^2+^-dependent disassembly of cortical F-actin via the action of scinderin.^49^ He et al. reported that in IECs, GSDMD was cleaved to a 13 kD NT fragment by caspase-3/7 following exposure to dietary antigens.^222^ This fragment, distinct from the 30 kD NT fragment, migrates to the nucleus and stimulates the transcription of CIITA and MHCII molecules, which leads to the apoptosis of Tr1 cells in the proximal small intestine. This process enables IECs to foster protective immune responses against pathogens while preserving immune tolerance to dietary antigens.^222^ In addition, GSDMD also functions in full-length form. Zhang et al. demonstrated that GSDMD could enhance the susceptibility of tumor cells to chemotherapy by inducing ER stress, rather than via pyroptosis.^286^ This mechanism involves the upregulation of eIF2α binding to p-ERK and promotes the phosphorylation of eIF2α and the induction of ER stress. Following the upregulation of activating transcription factor 4 (ATF4) protein level, a cascade of events is initiated, leading to the activation of apoptosis-related proteins, including C/EBP homologous protein (CHOP). This activation is proposed to correlate with the susceptibility of the tumor to therapeutic agents, potentially influencing drug response outcomes.^286^ Similarly, GSDMB can act through the full-length form. In IBD, epithelial-derived GSDMB modulates the phosphorylation of focal adhesion kinase, thereby enhancing the preservation and regeneration of epithelial tissue.

These studies further sophisticate our current comprehension of the pyroptosis-independent function of GSDMs across various physiological and cellular contexts and suggest potential risks of using GSDMs as a therapeutic target for anti-inflammatory drugs. A pivotal yet intriguing inquiry lies in understanding the diverse responses of various cell types to these structurally akin GSDM-NT pores or full-length GSDM. Future investigations in structural biology might provide insights into this matter. Moreover, we speculate the presence of additional proteins that collaborate in the distinct functionalities of the GSDM-NT pores, as suggested by ref. ^243^

## Regulation of gasdermins

### Transcriptional regulation of gasdermins

GSDMs are pivotal in orchestrating cell death and inflammatory responses. The expression level of GSDMs has a direct impact on cellular susceptibility to pyroptosis, where the key lies in whether the formation of GSDM pores is sufficient to overwhelm the repair mechanism of PM, thereby triggering pyroptosis. Thus, regulating the expression of GSDMs becomes an effective strategy to modulate cell death and cytokines release. As investigating into the transcriptional regulators of GSDMs and their participation in pathological conditions deepens, we have gained a preliminary understanding of the regulatory mechanisms of GSDMs at the transcriptional level,^80^ but further exploration is still needed. Currently, there is a preliminary understanding of the transcriptional regulation of GSDMD, however, little has been explored for other GSDMs.

Recent investigations have elucidated that in mouse macrophages, the manifestation of GSDMD is governed by interferon-regulated factor 2 (IRF2), which acts by selectively combining with the transcription initiation site of GSDMD.^80^ The absence of IRF2 does not entirely abrogate GSDMD expression but does lead to a marked reduction in GSDMD level, accompanied by decreased release of IL-1β and reduced cell mortality. Conversely, human monocytes do not rely on IRF2 for GSDMD expression regulation.^287^ Nonetheless, the current understanding of the regulatory mechanisms involving these transcription factors, and their potential interplay with cofactors, remains incomplete.

While the regulatory mechanisms that govern GSDMD expression in homeostatic conditions have been partially elucidated, the comprehension of its transcriptional control during inflammation is still lacking. Upon LPS stimulation, adipocytes engage in GSDMD-dependent pyroptosis, a process mediated by the NF-κB signaling cascade.^288^ In human septic neutrophils, GSDMD transcription is regulated by STAT3, which involves nuclear PD-L1 translocation.^289^ STAT3 also regulates the transcription of GSDMC, a process that necessitates the participation of nuclear PD-L1. Upon macrophage-derived TNF-α activation, caspase-8 cleaves GSDMC at the D365 site, generating GSDMC-NT, which ultimately leads to pyroptosis.^35^ Furthermore, the transcriptional activation of GSDMD in response to cytosolic *A. baumannii* infection Furthermore, the transcriptional activation of GSDMD in response to cytosolic A. baumannii infection relies on IRF3/7 and IFNAR1.^290^ Recently, it was found that the Sp1 positively modulates the transcriptional control of GSDME by binding −36–−28 sites in the GSDME promoter, and promotes the pyroptosis of tumor cells.^196^ A comprehensive investigation into the transcriptional control of GSDMs is anticipated to reveal novel therapeutic approaches for managing this pivotal protein family, and additional studies are needed to pinpoint the pathways that trigger GSDMs expression.

### Post-translational modifications of gasdermins

#### Ubiquitination of gasdermins

Ubiquitination, a pivotal post-translational modification, is integral to the “quantitative” and “qualitative” regulation of proteins in many biological and disease processes.^291–293^ The process consists of multiple enzyme-catalyzed stages, involving the coordinated activity of ubiquitin-activating enzymes (E1s) and ubiquitin-conjugating enzymes (E2s) to ubiquitin ligases (E3s), leading to the covalent attachment of ubiquitin to the target protein.^294–296^

Inflammasomes regulated by ubiquitination have been extensively studied^297–302^; however, ubiquitination on GSDMs has been reported less frequently, but recent studies have yielded intriguing insights into this process (Table 2). The human GSDMB and GSDMD are directly implicated in the lysis of incoming bacterial pathogens and the cells they have infected, whereas the bacterial E3 ubiquitin ligase IpaH7.8 can ubiquitinate degradation of human GSDMB and GSDMD, potentially enabling pathogen escape.^160,162^ The recently published structure for the GSDMB and IpaH7.8 LRR complex has provided valuable insight into the mechanism of this ubiquitination.^38,163^ The interaction between GSDMB-NT and the IpaH7.8 LRR is mediated by charged and hydrophobic residues, with specific GSDMB residues (E15, D21, L96D, R124, and R208) being essential for this association. It is worth mentioning that the binding of IpaH7.8 to GSDMs is not a universal precursor to ubiquitination or protein degradation. This is exemplified by the fact that IpaH7.8 binds both hGSDMD and mGSDMD proteins, yet it specifically ubiquitinates and degrades only the human protein, sparing the mouse equivalent.^38,162,163^ This feature may enable mice to capitalize on mGSDMD-induced pyroptosis as a defense mechanism against Shigella infection. Another report also shows that the E3 ubiquitin ligase SYVN1 engages with GSDMD, mediating the non-proteasomal polyubiquitination of Lys27-linked GSDMD at residues Lys203 and Lys204 in humans (Lys204 and Lys205 in mouse).^303^ Interestingly, this process promotes pyroptosis rather than inhibition, and the mechanism involved is not known.

**Table 2:** PTMs modulating the activities of the GSDM family

PTM	GSDM family	Modified residue	Effect	Ref.
Lys48-linked polyubiquitination (IpaH7.8)	GSDMB	At least Lys177, Lys190, Lys192 (human)	Promote degradation of GSDMB and directly inhibit pore formation	^38,160–163^
Lys63-linked/ Lys48-linked polyubiquitination (IpaH7.8)	GSDMD	LysK55, Lys62, Lys203 (human)	Promote degradation of GSDMD	^38,161–163^
Lys27-linked Polyubiquitination (SYVN1)	GSDMD	Lys203, Lys204/Lys204, Lys205 (human/mouse)	Promote pyroptosis	^303^
Lys48-linked Polyubiquitination (CDC20)	GSDME	Not mentioned	Promote degradation of GSMDE	^305^
Deubiquitination (USP24)	GSDMB	Not mentioned	Increase the stability of GSDMB	^304^
Deubiquitination (USP48 and OTUD4)	GSDME	Not mentioned	Increase the stability of GSDME	^306,307^
Phosphorylation (AMPK)	GSDME	Thr6 (human)	Prevent pore formation	^59,311^
Phosphorylation (PLK1)	GSDMA	Thr8 (human)	Prevent pore formation	^59,312^
Phosphorylation	GSDMD	Thr213 (human)	Prevent pore formation	^32^
Disulfiram	GSDMD	Cys191/Cys192 (human/mouse)	Prevent pore formation	^45^
Disulfiram	GSDME	Not mentioned	Prevent pore formation	^530^
Necrosulfamide	GSDMD	Cys191/Cys192 (human/mouse)	Prevent pore formation	^44^
Succination (fumarate)	GSDMD	Cys191/Cys192 (human/mouse)	Prevent cleavage and pore formation	^46^
Succination (fumarate)	GSDME	Cys45 (mouse)	Prevent pore formation	^46^
Itaconation	GSDMD	Cys77 (mouse)	Prevent caspase-1-dependent cleavage	^320^
Oxidation	GSDMD	Cys38, Cys56, Cys268, Cys467 (human)	Promote cleavage by caspase-1	^323^
Palmitoylation (ZDHHC5/9)	GSDMD	Cys191/Cys192 (human/mouse)	Promote pore formation and pyroptosis	^324,325^
Palmitoylation (ZDHHC-2/7/11/15)	GSDME	Cys407/Cys408 (human/mouse)	Promote pore formation and pyroptosis	^326^
Palmitoylation	bacterial and fungal GSDMs	Cys3/Cys3/Cys4/Cys7 (Runella/ Bradyrhizobium/ Vitiosangium/Lysobacter)	Promote structural stability and pore-forming activity	^228^

In addition, GSDMB and GSMDE are also regulated by ubiquitination. USP24 interacts with GSDMB and acts as a deubiquitinating enzyme (Dub) to remove polyubiquitin chains from GSDMB,^304^ increasing the stability of GSDMB in bladder cancer and further promoting downstream phosphorylation of STAT3, which promotes bladder cancer cell proliferation. Caspase-3/GSDME-dependent pyroptosis is a key determinant of anti-tumor immunity. The E3 ubiquitin ligase CDC20 reduces tumor cell pyroptosis through ubiquitinated degradation of GSMDE.^305^ The Dub USP48 and OTUD4 promote GSDME-mediated pyroptosis by deubiquitinating and stabilizing GSDME, which increases the sensitivity of tumor cells to treatment.^306,307^ It is clear that we are just beginning to understand the regulatory role of ubiquitination on GSDMs, and the mechanisms of GSDM recognition and ubiquitination remain to be elucidated.

#### Phosphorylation of gasdermins

Phosphorylation, as a pervasive protein modification mechanism, permeates numerous signaling processes and serves as a pivotal regulator across diverse levels of cellular activity.^308,309^ Evidence suggests that the operational dynamics of GSDMs may be fine-tuned by this post-translational modification, albeit the underlying mechanics remain largely elusive. Currently, the presence of phosphorylation has only been found in humans for GSDMA, GSDME and GSDMD (Table 2). Analysis of the PhosphoSitePlus mass spectrometry database^310^ revealed phosphorylation of GSDME at multiple serine (Ser) and threonine (Thr) sites, including Thr6, Ser69, Ser113, Ser114, Thr117, and Ser252.^59^ In particular, phosphorylation of Thr6 prevents the oligomerization of GSDME in the membrane and its induced pyroptosis,^59,311^ and a recent report elucidated that adenosine monophosphate-activated protein kinase (AMPK) acts as its kinase.^311^ Phosphorylation of the Thr8 site of GSDMA is mediated by polo-like kinase 1 (PLK1), a process that, similar to GSDME, prevents the ability of GSDMA from forming pores in PM.^59,312^ Both Thr8 and Thr6 are situated on the α1 helices of both GSDMA and GSDME, which helices are essential for protein oligomerization, and phosphorylation may produce charge repulsion, thereby hindering the assembly of GSDM pores.^313^ GSDMD-mediated pyroptosis is attenuated when Ser and Thr residues are converted to Glu residues.^314^ Gel electrophoretic migration analysis has uncovered that Thr213 serves as the principal phosphorylation site that inhibits GSDMD oligomerization, but the specific kinase is not known. Furthermore, it appears that only in their phosphorylated forms can GSDMD-NT and GSDMA-NT interact with membrane lipids, indicating that alterations in membrane lipid composition, coupled with phosphorylation, represent an additional regulatory mechanism within the pyroptosis process.^32^ As to whether the remaining GSDMs modulate their function through direct phosphorylation remains unresolved, although each of these proteins harbors at least one Ser or Thr residue within the α1 helix.

The phosphorylation of caspases interacting with GSDMs could potentially serve as a pathway for regulating the activity of GSDMs. Ser376 stands as the sole characterized phosphorylation site on caspase-1, which is essential for its activation.^315^ Furthermore, studies have demonstrated that the phosphorylation of caspase-3/7/8 modulates their activation processes and/or abilities to recognize substrates.^316–319^ However, the phosphorylation of caspase-4/5/11 has not been investigated, and a possible explanation is that they have a low activation threshold or do not require phosphorylation modification to trigger pyroptosis.

#### Cys modifications of gasdermins

In 2016, Liu et al. illuminated the essential function of Cys191 in hGSDMD and Cys192 in mGSDMD in orchestrating the pyroptotic response (Table 2).^30^ Subsequent investigations revealed that a mutation at Cys191 in hGSDMD can diminish the pyroptotic frequency by half.^45,50^ Meanwhile, small molecule compounds such as NSA^44^ and DSF^45^ have been shown to hinder hGSDMD-mediated apoptosis by covalently modifying Cys191. In addition, the metabolite fumarate irreversibly binds Cys191 of hGSDMD, Cys192 of mGSDMD, and Cys45 of mGSDME in a process known as succination,^46^ which is effective in inhibiting the cleavage and oligomerization of hGSDMD and the resulting cell death, and has demonstrated therapeutic effects in animal models of lethal endotoxemia. These insights suggest that metabolic shifts, particularly from oxidative phosphorylation to aerobic glycolysis, can regulate the formation of GSDM pores. Recent research further endorses the notion that metabolic transitions exert influence over GSDM-mediated pyroptosis. The accumulated cellular metabolite itaconic in macrophages that are challenged by prolonged LPS stimulation directly interacts with GSDMD at the Cys77 site, thereby preventing GSDMD pore mediated by caspase-1 and making the cells tolerant to prolonged LPS exposure.^320^ In addition, ROS, which is generated in response to inflammasome stimulation,^321,322^ can modulate the activity of hGSDMD by directly oxidizing Cys38, Cys56, Cys268, and Cys467 of hGSDMD, and mutation of these residues reduces GSDMD pore formation.^313,323^

Recent studies have revealed the mechanism by which Cys191/Cys192 (human/mouse) is essential for GSDMD pores. Post-translational palmitoylation of GSDMD at the Cys191/Cys192 site is a decisive step in the transfer of GSDMD-NT to PM for pore formation, which is facilitated by the palmitoyl acyltransferases ZDHHC5/9 and is potentiated by ROS induced by LPS.^324,325^ Inhibition of palmitoylation of GSDMD by using the palmitate analog 2-bromopalmitate significantly reduces macrophage pyroptosis and the secretion of IL-1β, thereby alleviating the pathological state in septic mice. In addition to GSDMD, Cys407/Cys408 (human/mouse) of GSDME are also palmitoylated during chemotherapy-induced pyroptosis, and ZDHHC-2/7/11/15 have been identified as the acyltransferases responsible for palmitoylating GSDME.^326^ This palmitoylation may aid in the separation of GSDME-NT and GSDME-CT without altering the caspase-3-mediated cleavage of GSDME, a process that can be inhibited by 2-bromopalmitate. In addition, palmitoylated cys residues have been found in bacterial and fungal GSDM homologs, contributing to structural stability and maintaining pore-forming activity in anti-phage defense.^228^ Protein palmitoylation, a widespread form of acylation, represents a fundamental regulatory mechanism governing membrane binding, localization, stability, and protein interactions.^327,328^ Collectively, Cys modification emerges as a conservative regulatory mechanism of paramount importance in controlling the function of GSDMs.

## Gasdermins and diseases

GSDMs have been initially characterized for their involvement in a spectrum of pathologies, encompassing hearing impairment,^16,20^ asthma,^151,212^ hair loss,^19,209^ and cancer.^146,153^ Despite years of investigation, the specific biological roles of these proteins have remained elusive. Nonetheless, researchers have proposed a connection between GSDMs and inflammation. For instance, mutations in the Gsdma3 gene have been associated with alopecia in mice, a condition that is characterized by the depletion of stem cells, hyperkeratosis, and concurrent inflammation.^329^ Presently, aggregation of GSDM pores within PM is considered to be a signature feature of pyroptosis. Although this notion somewhat restricts GSDMs primarily to their role in pyroptosis, contemporary research indicates that these proteins may also participate in diverse cellular death mechanisms^330^ and additional non-lytic pathways, which together mediate inflammatory progression. GSDMs have been involved in a wide array of pathologies, spanning sepsis, viral infections, cancers, cardiovascular diseases, neurodegenerative diseases, metabolic diseases, and autoimmune diseases.

### Sepsis

Sepsis is considered a dysregulated immune response to pathogenic challenge, leading to profound and potentially fatal injury to tissues and organs.^331–335^ The etiology of sepsis is multifaceted and involves numerous aspects of the interaction between the invading microorganism and the host, including persistent excessive inflammation and immunosuppression, as well as an inability to restore homeostasis.^336–341^ Inflammatory imbalance is the most critical basis underlying the pathogenesis of sepsis, persisting throughout the progression of the disease. GSDMD is a critical regulator of pro-inflammatory cytokine secretion by immune cells, and recent evidence suggests a pivotal modulatory role for GSDMD in the pathogenesis of sepsis.^241,342^ Although various sensors and mediators activate pyroptosis, the pore-forming activity of GSDMD-NT emerges as a compelling therapeutic target, as it is a universal terminal step required for pyroptosis and the secretion of pro-inflammatory cytokines in response to pathogenic or danger-induced signals. GSDMD inhibition or inactivation does prevent lethal bacterial sepsis, with GSDMD^−/−^ mice displaying markedly enhanced survival over WT controls in models of sepsis induced by LPS and cecum ligation and puncture (CLP).^7,28,29,180,343–345^ GSDMD serves as a crucial mediator in macrophage pyroptosis and the secretion of IL-1β, a classic pro-inflammatory cytokine that initiates the host inflammatory response and amplifies both innate and adaptive immune responses.^74^ Excessive and persistent IL-1β secretion plays a significant role in the systemic inflammation and organ damage characteristic of severe sepsis,^60,346–348^ but two phase III clinical trials could not yield beneficial effects of anti-IL-1β receptor antibodies in septic patients.^349,350^ Consequently, inhibiting GSDMD-mediated cytokine production may represent an effective strategy for treating sepsis. For instance, GSDMD^−/−^ mice are protected against lethal septic shock induced by LPS.^28^ Kayagaki et al. reported that BMDMs lacking GSDMD are insusceptible to pyroptosis and do not release IL-1β in response to transfection with LPS or synthetic monophosphoryl lipid A. Similarly, GSDMD^−/−^ BMDMs were unresponsive and failed to produce IL-1β upon electroporation with LPS, or when LPS was complexed with cholera toxin B subunit or stimulated with *S. typhimurium* LPS.^28^ Consequently, targeting GSDMD inhibition emerges as a promising strategy to mitigate inflammation, as Hu et al. reported that the FDA-approved alcoholism treatment drug DSF effectively inhibited GSDMD pore formation, thereby blocking LPS-induced septic death.^45^ DSF-induced modification of Cys191 in hGSDMD (Cys192 in mGSDMD) is required to mediate conformational changes in membrane insertion and pore formation. DSF preserves the processing of IL-1β and GSDMD but inhibits the formation of pores, thereby inhibiting the release of IL-1β and the execution of pyroptosis.^45^ Notably, IL-1β is not uniformly detrimental due to the different triggers of sepsis. Indeed, IL-1β increased host resistance against *C. albicans* and mitigated diffuse infections caused by this pathogen.^351–353^ Surprisingly, GSDMD^−/−^ mice are resistant to *C. albicans* infection and accompanying kidney injury, partly because IL-1β does not rely on GSDMD release to generate antifungal host defense. Another reason is that GSDMD inhibition prevents the escape of *C. albicans* and promotes the clearance of the fungus.^352^

Coagulation dysfunction stands as a prevalent and severe manifestation of sepsis, occasionally resulting in disseminated intravascular coagulation (DIC), which is a pathological state marked by systemic thrombotic activation, microvascular occlusion, organ dysfunction, and death.^172,354–356^ Abnormalities in the coagulation system play a contributory role in the onset of sepsis, where GSDMD has recently been found to play an important role.^357–360^ Tissue factor (TF), a critical initiator of the coagulation cascade, plays a decisive role in triggering systemic DIC.^361,362^ GSDMD knockout attenuates LPS-induced DIC, including blocking thrombin production, fibrin formation, platelet accumulation and microvascular occlusion in the liver, as well as increases in plasma thrombin-antithrombin (TAT), D-dimer and plasminogen activator inhibitor type-1 (PAI-1).^357^ Yang et al. documented that caspase-11 triggered GSDMD pores assembly, facilitated Ca^2+^ influx, and led to phosphatidylserine exposure via the activity of transmembrane protein 16 F, an enzyme involved in Ca^2+^-mediated phospholipid scrambling. Independent of pyroptosis, enhanced activation of TF promoted the development of DIC.^357^ As plasma IL-1β concentrations correlate with DIC scores in patients with sepsis, this study suggests that caspase-11/GSDMD signaling may offer new therapeutic avenues for sepsis-associated DIC. Zhang et al. similarly suggested the crucial role of GSDMD in lethal septic DIC.^360^ TMEM173-dependent increase in cytoplasmic Ca^2+^ drives GSDMD cleavage, thereby initiating the delivery of F3, a critical activator in the blood coagulation cascade. The procoagulant and lethal effects elicited by CLP-, *E. coli*-, or *S. pneumonia* are inhibited in mice harboring a mutated GSDMD cleavage site or through the administration of anti-F3 antibodies. Furthermore, platelets, which significantly contribute to DIC, have recently been shown to undergo GSDMD-induced pyroptosis, exacerbating the formation of NETs and inflammation during sepsis. Su et al. demonstrated that platelet pyroptosis fostered inflammation and multi-organ damage in CLP-induced sepsis using platelet-specific GSDMD knockout mice.^363^ Pyroptotic platelets may release oxidized mitochondrial DNA (ox-mtDNA), which promotes NET formation, exacerbating platelet pyroptosis through the release of S100A8/A9 that targeted toll-like receptor 4 (TLR4), creating a self-amplifying cycle that results in excessive cytokines release.

Neutrophils constitute the predominant circulating white blood cells and function as the front-line guardians of the host immune response against invading pathogens.^260,277,364–366^ During sepsis, activated neutrophils release NETs - complex webs of DNA ensnaring antimicrobial proteins—to facilitate the destruction of pathogens.^276^ However, an increasing array of findings underscores the detrimental impact of NETs in the progression of sepsis.^277,367,368^ Recently, evidence has emerged indicating that GSDMD is implicated in the formation and release of NETs^42,43^ and the development of sepsis.^52,289^ Silva et al. discovered that GSDMD^−/−^ mice exhibited significantly diminished intravascular NET levels in CLP-induced sepsis, with parallels observed in vitro, where cytosolic LPS failed to prompt NET formation by GSDMD^−/−^ neutrophils.^52^ GSDMD^−/−^ mice exhibited decreased levels of inflammatory cytokines, improved organ dysfunction, and increased survival in the CLP model. Similar protective effects were observed in WT mice treated with DSF. Transfer of GSDMD-expressing WT neutrophils into GSDMD^−/−^ mice reversed the protective effect of the organs against sepsis and elevated serum NET levels. Septic neutrophils from patients undergoing NETosis display GSDMD expression on PM and are correlated with the formation of prototypical NET structures.^52^ In addition, our team demonstrated the deleterious role of GSDMD-mediated NET release in sepsis-associated encephalopathy (SAE), and neutrophil-specific GSDMD knockout reduced plasma and hippocampal NET levels as well as ameliorated inflammatory injury in a murine model of SAE.^289^ Neutrophil PD-L1 can be translocated to the nucleus, aided by the help of p-Y705-STAT3, to constitute the nPD-L1/p-Y705-STAT3 complex, which promotes the transcription of GSDMD. Consequently, they and we propose that therapeutically targeting GSDMD to directly inhibit NETosis, or targeting upstream regulators of GSDMD to indirectly inhibit NETosis, may represent an efficacious strategy for the treatment of sepsis. In contrast, Liu et al. reported that the occurrence of sepsis induced by CLP in neutrophil-specific GSDMD knockout mice strikingly increased inflammatory cytokine levels, promoted tissue damage, and reduced survival, suggesting that the absence of neutrophil GSDMD does not provide protection against polymicrobial sepsis but rather predisposes mice to a more severe manifestation of the disease.^223^ This is contrary to the findings of previous studies.^52,289^ They agree that systemic ablation of GSDMD confers protection against lethal sepsis in mice and that NET release from GSDMD^−/−^ neutrophils in vitro is indeed reduced, but they also confirm that neutrophil-specific GSDMD knockout mice have higher inflammatory cytokines, higher bacterial loads, and higher mortality rates, as well as no reduction in NET levels in vivo.^223^ They suggest that depletion of GSDMD in neutrophils may impair their bactericidal activity, so that neutrophils lose the ability to remove replicative ecological niches of pathogens, and pathogens are no longer readily engulfed and killed by secondary phagocytes. This exacerbates infections, which subsequently triggers an increased production of cytokines by myeloid cells, resulting in hyperinflammation in CLP mice. The establishment of a GSDMD-dependent positive feedback loop involving platelets and NETs proposed by ref. ^363^ may be an explanation for the absence of reduced NETs in neutrophil-specific GSDMD knockout mice. This implies that in addition to regulating NETs, GSDMD may be involved in sepsis through other mechanisms. Recently, Pruenster et al. discovered that E-selectin triggers the prompt secretion of S100A8/S100A9 from neutrophils through a reversible activation mediated by the NLRP3/GSDMD axis.^369^ This rapid activation process is not dependent on the involvement of TLR4, and is followed by the prompt assembly of the ESCRT-III PM repair mechanism, which coincides with the rapid formation of GSDMD pores mediated by E-selectin. Neutrophils may be involved in sepsis through the mechanisms dependent on GSDMD but unrelated to NETs. Collectively, these investigations underscore that GSDMD indeed plays a crucial role in sepsis pathology. Nonetheless, the function of GSDMD in pyroptosis, NET release, and the onset of sepsis is complex, necessitating additional studies to comprehensively delineate its mechanisms of action.

### Virus infection

With the growing understanding of pyroptosis, the phenomenon that various virus infections can trigger pyroptosis has come to the forefront. During human adenovirus (HAdVs) infection, the HAdVs genome 36 kb dsDNA is detected by AIM2, which initiates the assembly of an inflammasome complex. Subsequent caspase-1 activation, GSDMD cleavage, and IL-1β release result in the pyroptotic death of human monocyte-derived dendritic cells (MoDCs),^370^ which is a pivotal component of the innate immune response elicited by viral infection. During human norovirus (HuNoV) infection, the nonstructural protein P22 activates the NLRP3 inflammasome in enteric stem cell-derived human intestinal enteroids (HIEs), contributing to pyroptosis, and GSDMD pore-released IL-1β and IL-18 promotes inflammation in virus infections.^371^ Rotavirus infection also leads to pyroptosis in IECs. Specific expression of a novel NLR inflammasome in IECs, NLRP9b, recognizes short dsRNA stretches by the RNA helicase Dhx9, assembling an inflammasome containing the adapter protein ASC and the cysteine protease caspase-1 to promote GSDMD-induced pyroptosis and IL-18 release.^372^ This is particularly critical for host defense to limit rotavirus replication by triggering the premature death of infected IECs while preserving gut homeostasis. Previously, the lethal attack of human immunodeficiency virus (HIV) on its primary cellular target, CD4 T cells, was usually attributed to apoptosis. It is now believed that caspase-1-mediated pyroptosis appears to be the primary cause of CD4 T cell death driven by HIV infection of lymphoid tissues. This results in substantial secretion of IL-1β, which may further exacerbate chronic inflammation.^373–375^ In addition, intestinal mucosal-associated invariant T (MAIT) cells from patients infected with HIV-1 show robust GSDMD-driven pyroptotic signals adjacent to the luminal side, indicating that MAIT cells undergo pyroptosis within the colorectal mucosa, which promotes an increase in inflammatory cytokines and may exacerbate disease progression and hinder effective immune reconstitution.^376^ It comes as no surprise that GSDMD is implicated in the progression of virus infections as a prominent agent of pyroptosis, while IL-1β and IL-18 contribute to antiviral immunity. Notwithstanding, the function of GSDMD in virus infections remains obscure, despite extensive research into its regulatory functions within the inflammasome framework in response to cytosolic bacteria or LPS activation.

Recently, with the outbreak and epidemic of COVID-19, the study of coronavirus pathogenesis has deepened, and the significance of GSDMD in virus infections has gained new understanding. SARS-CoV-2, the virus linked to COVID-19, is an enveloped RNA virus comprising multiple proteins, including nucleocapsid, matrix, envelope, and spike.^377–379^ COVID-19 typically presents as a respiratory disease with severe inflammation of the lungs in critically ill individuals, potentially leading to multi-organ dysfunction and mortality in geriatric and comorbid patient populations.^380,381^ According to Junqueira et al., ~10% of monocytes and 8% of lung macrophages from individuals with COVID-19 were found to be infected with SARS-CoV-2, and pyroptosis pathways were activated, contributing to cell death and inflammatory mediators release, which in turn caused cytokine storms.^382^ This study also suggests that the internalization of virus-antibody complexes by monocyte-dependent Fcγ receptor results in GSDMD-dependent pyroptosis, which potentially represents a substantial mechanism underlying the severe inflammatory sequelae, leading to vascular leakage, acute lung injury, and multi-organ damage in severe cases.^382^ Two currently FDA-approved GSDMD inhibitors, DSF (Antabuse)^45^ and dimethyl fumarate (DMF, tecfidera),^46^ are undergoing evaluation in clinical trials to assess their protective effects against COVID-19 (NCT04485130, NCT04594343, and NCT04381936), which further indicates that inhibition of GSDMD in the COVID-19 may be of therapeutic significance (Table 3). The researchers did not detect infected neutrophils in COVID-19 patients, suggesting that neutrophil infection may not be a central mechanism in pathogenesis, although NETosis induced by GSDMD may be an essential driver.^382^ Conversely, Silva et al. reported a significant role for GSDMD-dependent NETosis in the immunopathology of COVID-19, proposing that interventions targeting GSDMD could represent a novel strategy for enhancing therapeutic approaches to the disease.^383^ They observed that serum NET and GSDMD levels were elevated and positively correlated with severe cases of COVID-19. The activation of GSDMD-mediated NET in neutrophils requires caspase-1/4 and SARS-CoV-2, which can be abrogated by DSF treatment. In a mouse model infected with SARS-CoV-2, DSF treatment inhibits NET release and attenuates lung damage.^383^ Similarly, our team demonstrated that peripheral blood neutrophil NET release correlated with GSDMD in patients experiencing acute respiratory distress syndrome (ARDS).^384^ Using an intratracheal LPS-induced mouse model of ARDS, lung NET accumulation and ARDS injury were significantly attenuated in neutrophil-specific GSDMD knockout mice or DSF-treated WT mice, demonstrating a significant association between the progression of lung injury in ARDS and the accumulation of NETs mediated by GSDMD. In combination with the study of ref.,^52^ GSDMD-induced NETosis emerges as a pivotal mechanism in the development of lung injury. Moreover, Ma et al. illustrated that after infection, the nucleocapsid of SARS-CoV-2 could inhibit host pyroptosis and counteract cellular inflammatory responses by blocking the cleavage of GSDMD.^385^ The nucleocapsid protein of SARS-CoV-2 binds to the GSDMD linker region in infected monocytes and hinders caspase-1-mediated processing of GSDMD, restraining GSDMD cleavage and leading to reduced IL-1β secretion, despite enhanced IL-1β expression at this time. This further explains the close association of GSDMD with anti-SARS-CoV-2 activity, as the virus has also evolved this mechanism to avoid GSDMD cleavage.

**Table 3:** FDA-approved drugs and clinical trials related to gasdermins

Clinical trial	FDA-approved drugs	Dose and schedule	Indication	Enrollment	Study Start/ Completion
NCT04594343 (phase 2)	Disulfiram	500 mg disulfiram orally or enterally daily for 14 days	Hospitalized subjects over the age of 50 with a diagnosis of moderate COVID-19	140	2020-11-20/2021-09-25
NCT04485130 (phase 2)	Disulfiram	Oral disulfiram for 5 consecutive days (cohort 1, 1000 mg/day; cohort 2, 2000mg/day)	COVID-19 patients with early mild to moderate symptoms	11	2021-08-18/2022-02-28
NCT04381936 (phase 2/3)	Including but not limited to dimethyl fumarate	Not mentioned	COVID-19 Inpatients	50,000 (estimated)	2020-03-19/ 2032-11 (estimated)

In NCT04381936, dimethyl fumarate has been shown to have no beneficial effect on COVID-19 inpatients and recruitment for this drug has been stopped

In addition to the widely publicized SARS-CoV-2, the function of GSDMD in swine enteric coronavirus infections has been tentatively explored, such as enteric coronavirus transmissible gastroenteritis virus (TGEV), porcine delta coronavirus (PDCoV), and porcine epidemic diarrhea virus (PEDV). TGEV and PDCoV upregulate and activate GSDMD, leading to post-infectious pyroptosis.^386^ Knockdown of GSDMD or pharmacological inhibition of GSDMD reduces IFN-β release, suggesting that GSDMD is associated with its ability to facilitate the non-conventional secretion of IFN-β, which enhances the IFN-stimulated gene (ISG) response. The 3C-like protease Nsp5 of PEDV is capable of cleaving porcine GSDMD at the Q193-G194 site to generate two fragments that lack the capacity to initiate pyroptosis, thereby promoting the propagation of the virus at the initial stage and sustaining PEDV infection.^387^ Notably, GSDMD also serves a critical role in non-coronavirus infection. The protein S273R encoded by the African swine flu virus (ASFV) specifically cuts GSDMD at the G107-A108 site, producing a short segment of the GSDMD-NT domain (GSDMD-N1-107) composed of residues 1 to 107, which fails to activate pyroptosis or curtail the replication of ASFV.^388^ When infected with enterovirus 71 (EV71), the viral protease 3C specifically targets Q193-G194 sites on GSDMD, facilitating proteolytic cleavage, which is protease-dependent and produces a short N-terminal segment across aa 1–193 (GSDMD_1-193_) that lacks the ability to elicit cell death or impede the replication of EV71.^389^ These studies reveal a possible mechanism by which ASFV and EV71 evade antiviral responses.

Excessive inflammatory response and damage to tissues under influenza virus attack may progress to severe lung disease.^390^ Influenza A virus (IAV) triggers activation of GSDMD in lung epithelial cells, exacerbating pathological changes in the lungs and accumulation of immune cells. GSDMD^−/−^ mice show greater resistance to IAV infection, as evidenced by attenuated neutrophil recruitment and chemotaxis, reduced epithelial damage and cell death, and increased survival.^391,392^ In addition, the H7N9 influenza virus is able to activate GSDME, leading to pyroptosis of alveolar epithelial cells and triggering cytokine storms in the lung.^393^ Through the targeted deletion of GSDME, the cell death mechanism in alveolar epithelial cells infected with the H7N9 virus is transformed from pyroptosis to apoptosis. GSDME^−/−^ mice results in a notable reduction in lung inflammation and a substantial increase in survival rates when exposed to a lethal dose of the H7N9 virus.^393^ Recently, activation of GSDME has also been found to be associated with Zika virus (ZIKV), foot-and-mouth disease virus (FMDV), and oncolytic parapoxvirus ovis (ORFV) infections. ZIKV activates GSDME via TNF-α/caspase-8/caspase-3, causing a significant increase in GSDME activation and in placental cell pyroptosis.^394^ FMDV 3Cpro cleaves the Q271-G272 junction of porcine GSDME to trigger pyroptosis, a pathway that is not contingent upon caspase-3.^395^ ORFV attack activates GSDME-induced pyroptosis by decreasing the ubiquitination of GSDME.^396^ Reducing the expression of GSDME both reduces these virus-induced pyroptosis and improves the disease phenotype.

The above findings hint that the pore-forming activity of GSDMs can effectively inhibit virus replication and immune escape in various virus infections, thus accounting for the evolutionary adaptation of viruses to deploy various strategies to evade GSDM activation. GSDMs may represent a novel therapeutic candidate for refining the management of virus infections, but the precise mechanisms require comprehensive further investigation.

### Cancers

There is mounting evidence supporting the potential involvement of GSDMD in diverse cancers. NLRP3/GSDMD-dependent pyroptosis pathway has been implicated in the progression of cancer, including non-small cell lung cancer (NSCLC),^397,398^ triple-negative breast cancer (TNBC),^399^ ovarian cancer,^400^ and colorectal cancer.^401,402^ GSDMD is observed in differentiated cells of gastric cancer (GC) and exhibits colony formation inhibitory activity, potentially inhibiting cell proliferation.^153^ GSDMD is also implicated in the invasive and metastatic potential of colorectal cancer cells and is a negative regulator.^403^ Moreover, GSDMD-mediated pyroptosis has an immunostimulatory effect, being essential for enhanced spontaneous anti-tumor immune responses and increased sensitivity to anti-PD-1 blockade in mixed-lineage leukemia 4 (Mll4)^−/−^ melanoma.^404^ The underlying mechanism of GSDMD-mediated anti-tumor activity has been partially elucidated. According to Wang et al., silencing GSDMD expression in gastric cancer (GC) cells enhanced their proliferation and tumourigenesis in nude mice. This downregulation activated the PI3K/AKT, STAT3, and ERK1/2 signaling cascades, which in turn modulated the expression of CDK-2 and cyclin A2, leading to an acceleration of the S/G2 cell cycle transition. These findings suggest that GSDMD functions as a suppressor of GC cell proliferation.^405^ Xi et al. demonstrated that GSDMD facilitates the cytotoxic activity of T lymphocytes (CTLs) against cancer cells, primarily by delivering the contents of cytotoxic granules into the immune synapse established with the tumor cells.^406^ Elevated levels of GSDMD processing within CTLs, concomitant with the proximity of GSDMD to granzyme B, are detected in the perimeters of the immune synapse, and GSDMD knockdown decreases cytotoxicity of CTLs. They propose that GSDMD may be essential for the robust activation of CTLs against cancer cells, although the role of GSDMD in CTL remains undefined.^406^ Remarkably, GSDMD also exhibits pyroptosis-independent roles in the context of cancer. Peng et al. suggested that under specific stress conditions, such as hypoxia or cytotoxic treatment, GSDMD is directed to the nucleus to promote apoptosis, which has been correlated with positive clinical outcomes in cases of colorectal cancer. After nuclear translocation, GSDMD engages in a complex with poly (ADP-ribose) polymerase 1 (PARP-1), significantly suppressing the function of PARP-1 on DNA damage repair, thereby functioning as a tumor suppressor to enhance apoptosis in cancer cells.^407^ They concluded that the subcellular distribution of GSDMD could potentially help guide the treatment of colorectal cancer.

Conversely, GSDMD is prominently enhanced in NSCLC and is thought to initiate cancer. Knockdown of GSDMD inhibits cancer growth in vivo and in vitro, concurrent with the activation of caspase-3 and PARP cleavage, and enhances cancer cell death via the mitochondrial intrinsic apoptotic pathway.^408^ Furthermore, high GSDMD expression in lung adenocarcinoma (LUAD) implies a poor prognosis relative to lung squamous cell carcinoma (LUSC). Lv et al. identified high GSDMD expression as a promoter of hepatocellular carcinoma (HCC) development.^409^ The HMGB1/TLR4/caspase-1 pathway is involved in the upregulation and processing of GSDMD. Cyclic GMP-AMP synthase (cGAS) activation is inhibited by GSDMD-NT through the efflux of K^+^ to promote autophagy, and by histone deacetylase/STAT1 through the influx of Ca^2+^ to induce transactivation of PD-L1 to promote PD-L1 expression. GSDMD^−/−^ or WT mice treated with a combination of the GSDMD inhibitor DMF and an anti-PD-1 antibody showed reduced liver tumors and decreased PD-L1 expression.^409^ Consequently, the authors proposed that an approach encompassing both anti-PD-1 and GSDMD inhibitors could be effective in treating HCC with upregulated GSDMD. Similarly, a combined GSDMD/PD-L1 suppressive immunotherapy in improving anti-tumor immunity was also suggested by Jiang and colleagues.^410^

GSDMD is enriched in TME antigen-presenting cells (APCs) and is associated with immune checkpoint characteristics. By conditionally deleting GSDMD, Jiang et al. demonstrated that GSDMD within APCs limited anti-tumor immunity when PD-L1 was inhibited, suppressed ISG expression through targeting the cGAS signaling, and thus inhibited the capacity of macrophages and DCs in presenting tumor-related antigens as well as CD8 T cell activity. Pharmacological inhibition of GSDMD with DMF in conjunction with anti-PD-L1 treatment markedly reduces tumor load and improves survival in melanoma mice.^410^ These studies provide new insights for combination therapy for cancers that, while anti-PD-1/anti-PD-L1 therapy is effective, numerous patients do not respond to this treatment.

IL-33 is recognized as a tumor-promoting cytokine, and Yamagishi et al. elucidated the mechanism underlying which IL-33 was exported from senescent hepatic stellate cells (HSCs) through the GSDMD pores in a mouse model of HCC induced by obesity.^224^ In the tumor microenvironment, caspase-11 cleavage is induced by lipoteichoic acid (LTA) in senescent HSCs, and GSDMD-NT forms pores in PM, releasing IL-33 and IL-1β. IL-33 cleaved by elastase CELA1 promotes the development of HCC through the activation of ST2-positive T_reg_ cells.^224^ DSF treatment markedly curtails the secretion of IL-1β and IL-33 and suppresses hepatic tumor formation, suggesting the potential of inhibitors targeting the pore-forming process of GSDMD in the treatment of HCC.

Other GSDMs have been shown to be potentially associated with a multitude of cancers.^411,412^ Analyses of multiple bioinformatics databases demonstrate the involvement of GSDMs in HCC and clear cell renal cell carcinoma (ccRCC), with increased expression of GSDME correlating significantly with reduced overall survival in HCC and ccRCC patients.^411,412^ Studies have highlighted the engagement of GSDME in the pyroptotic demise of melanoma cells. Inhibition of eukaryotic elongation factor-2 kinase (eEF-2K) can inhibit autophagy and promote GSDME-mediated pyroptosis, which in turn modulates the susceptibility of melanoma cells to doxorubicin.^413^ ROS in the presence of iron have been implicated in triggering pyroptosis in melanoma cells via the Tom20-Bax-caspase-GSDME pathway.^187^ Inducing tumor pyroptosis to promote anti-tumor immunity is a potential cancer treatment strategy. Various drugs for cancer treatment act in part through caspase-3/GSDME-mediated tumor cell pyroptosis, including triptolide,^414^ mesothelin-targeting antibody-drug conjugate,^415^ apoptin,^416^ platinum-based drugs,^417,418^ tetraarsenic hexoxide,^419^ and alantolactone.^420^ GSDME knockout or knockdown may mitigate the anti-tumor potency of these agents. Similarly, GSDME-mediated pyroptosis also determines the effectiveness of radiotherapy for cancer treatment.^421^ Remarkably, cancer cells also use GSDME for their own survival strategies. In pancreatic ductal adenocarcinoma (PDAC), cells deploy GSDME to enhance mucin 1 and mucin 13 secretion, effectively establishing a protective barrier against the digestion enzyme chymotrypsin.^422^ This regulatory function of GSDME is distinct from its pyroptosis-inducing function, instead involving a regulatory mechanism where it interacts with and facilitates the nuclear translocation of the transcription factor Y-box-binding protein 1 (YBX1), which then directly enhances the expression of mucins.

In addition, researchers have uncovered a pivotal role for the post-translational modifications of GSDME in therapeutic interventions. Examination of multiple prostate cancer cohorts reveals that CDC20 interacts with GSDME and undergoes ubiquitination-mediated protein hydrolysis to negatively regulate tumor cell pyroptosis.^305^ The CDC20 small molecule inhibitor apcin exhibits synergistic effects with anti-PD-1 immunotherapy. The Dub USP48 promotes pyroptosis and enhances anti-tumor immunity by stabilizing GSDME. Mechanistically, USP48 binds GSDME and removes the k48-linked ubiquitination marks at positions K120 and K189. Pharmacological modulation of USP48 could represent a potent approach to trigger tumor cell pyroptosis.^307^ Similarly, OTUD4 deubiquitinates and stabilizes GSDME to enhance the sensitivity of nasopharyngeal carcinoma to radiotherapy by promoting pyroptosis.^306^ These insights underscore the critical function of pyroptosis in the body’s offensive against tumors. However, the very mechanisms that make pyroptosis a potent anti-tumor weapon—its ability to eliminate tumor cells—also pose a challenge. The activation of this pathway by chemotherapeutic agents can lead to unwanted collateral damage to healthy tissues. Ai et al. reported that during chemotherapy, mannose activates AMPK to inhibit GSDME-mediated pyroptosis, exerting a protective effect in the kidney and small intestine. Activated AMPK subsequently phosphorylates the Thr6 site of GSDME, thereby blocking the cleavage of GSDME induced by caspase-3 and thus inhibiting pyroptosis.^311^ This provides a new target for mitigating adverse reactions induced by chemotherapy in the clinic.

Downregulation of GSDMA significantly enhances the proliferation and invasive potential of esophageal cancer cells, a phenomenon that is intricately linked to changes in cell sensitivity to cisplatin.^423^ In individuals with HER2^+^ breast cancer, elevated levels of GSDMB correlate with reduced survival rates and an increased propensity for metastatic progression.^165,166^ It is shown that upregulation of GSDMB can confer resistance to therapeutic interventions in HER2^+^ cancer cells through activation of the protective autophagy pathway, in which the interaction of GSDMB-NT with LC3B and Rab7 is critical for the activation of pro-survival autophagy.^424^ Intracellular delivery of antibodies targeting GSDMB using hyaluronan-coated nanoparticles reduces the invasiveness of HER2^+^ breast cancer.^425^ Similarly, GSDMC may act as an oncogene, and its expression is upregulated in lung adenocarcinoma, melanoma, and colorectal cancer, promoting tumor progression and spread.^17,168,426^ However, it has also been suggested that GSDMC-mediated pyroptosis can exert anti-tumor activity.^35^ Collectively, the above findings suggest that GSDMs function in the development of various cancers, whether it plays an inhibitory or promotional role. The role played by GSDMs in cancer varies according to the environment, varying among different GSDM isoforms and cancer entities, underscoring the complex interplay between inflammation and tumourigenesis, cell proliferation, as well as anti-tumor immune responses.^427,428^ Further understanding of the functions of GSDMs, both pyroptosis-dependent and pyroptosis-independent, as well as its role in tumor immune checkpoints such as PD-1/PD-L1, may potentially impact the combination therapy strategies for tumor.

### Cardiovascular diseases

Cardiovascular diseases have also been linked to inflammasome activation and GSDMD-mediated pyroptosis.^429,430^ AS is a chronic condition characterized by dysregulated inflammation, lipid accretion, plaque development, and intimal hypertrophy, with a complex pathogenesis in which inflammation is fundamentally involved in the formation of AS.^431,432^ Inflammation inhibits reverse cholesterol transport (RCT) to promote AS, and interventions targeting IL-1β have demonstrated potential in mitigating cardiovascular disease risks in clinical settings.^433^ Opoku et al. suggested that GSDMD inhibited RCT and promoted AS in hyperlipidemic mice, and the possible mechanism is that macrophage GSDMD accelerated the formation of foam cells through an IL-1β-dependent manner. GSDMD^−/−^ macrophages maintain high cholesterol efflux activity through reducing IL-1β release and translocation of phosphatidylinositol 4,5-bisphosphate (PI(4,5)P(2)) to the cell surface, as well as reducing pyroptosis, and potentially tipping the equilibrium toward a more beneficial apoptotic cell death pathway.^434^ In addition, macrophage GSDME is involved in the pathogenesis of AS. ox-LDL stimulates GSDME expression, possibly by a mechanism whereby STAT3 binds to the GSDME promoter and activates its transcription. Subsequently, caspase-3-mediated cleavage of GSDME promotes pyroptosis and inflammation. GSDME deficiency attenuates macrophage pyroptosis and AS lesions.^195^

Not only macrophage pyroptosis is implicated in the initiation and advancement of AS, but endothelial dysfunction due to endothelial cell pyroptosis is also part of the pathogenesis of AS. For example, ox-LDL-induced upregulation of Hsa_circ_0090231 (circ-USP9×) levels within endothelial cells cytoplasm leads to pyroptosis. The interaction between circ-USP9× and EIF4A3 in the cytoplasm enhances the stability of GSDMD mRNA, which increases GSDMD expression and promotes endothelial cell pyroptosis. Knockdown of circ-USP9× expression using siRNA inhibits pyroptosis through eukaryotic initiation factor 4A-III (EIF4A3)-mediated GSDMD.^435^ Fan et al. reported that in endothelial cells, activation of the non-canonical NF-κB pathway triggers GSDMD-driven pyroptosis, promoting the development of AS.^436^ NLRP3 inflammasome signaling activates the non-canonical NF-κB transcription factor complex RelB/p52 to potentiate the expression of IRF1. IRF1 interacts with the GSDMD promoter-526/515 sites and caspase-1 promoter-11/10 sites to enhance the expression of GSDMD and its activation mediated by caspase-1.

Abdominal aortic aneurysm (AAA) is a prevalent vascular condition marked by cellular physiological modifications driven by active metabolites.^437,438^ Gao et al. demonstrated that vascular smooth muscle cell (VSMC)-specific GSDMD defects reduced the incidence of AAA in a mouse model.^439^ Mechanistically, GSDMD enhances ER stress-CHOP signaling, which subsequently stimulates the expression of ornithine decarboxylase 1 (ODC1), an enzyme that mediates an increase in putrescine levels. High putrescine triggers a pro-inflammatory switch in VSMCs and increases the vulnerability of mice to the development of Ang II-induced AAA. This reveals that GSDMD affects VSMC activity through a novel mechanism that is independent of pyroptosis, as GSDMD siRNA does not alter LDH release.^439^ They suggest that targeting GSDMD and putrescine may represent a novel therapeutic avenue for the treatment of AAA.

Cardiomyocyte injury can result from numerous factors, including endotoxin-induced inflammation, myocardial infarction (MI), ischemia/reperfusion (I/R), and doxorubicin (Dox) administration.^440^ In LPS and Nigerian bacteriocin-stimulated cardiomyocytes, Yu et al. suggested that GSDMD-NT translocated from mitochondria to cytoplasmic membranes in a time-dependent manner.^56^ In mitochondria, GSDMD-NT is capable of binding to LC3B, and GSDMD-induced mitochondrial damage results in inhibition of autophagic fluxes. Enhanced mitophagy in GSDMD^−/−^ cardiomyocytes provides protection against LPS-induced cardiomyocyte damage. They propose that GSDMD-NT-induced mitochondrial injury could be attenuated by mitophagy-mediated mitochondrial quality control, and that inhibition of GSDMD or enhancement of autophagy might serve as viable therapeutic targets for the amelioration of inflammatory cardiopathy.^56^

MI remains a significant contributing factor to global mortality.^441^ Although the prompt reinstatement of blood supply to ischemic myocardial tissue effectively reduces infarct size in MI patients, the benefits of reperfusion therapy are potentially attenuated by the deleterious effects of myocardial I/R injury.^442^ The efflux of cytokines from pyroptotic cardiomyocytes has the capacity to stimulate innate immune signaling pathways and trigger a potent inflammatory reaction. Shi et al. identified the caspase-11/GSDMD pathway, but not the caspase-1/GSDMD pathway, as a critical event in MI injury.^442^ Cardiac-specific GSDMD knockout significantly reduces myocardial infarct size in a mouse I/R model. Unexpectedly, oxidative stress-induced cardiomyocyte pyroptosis releases IL-18 rather than IL-1β. They and Kawaguchi et al. suggested that IL-1β expression originated mainly from fibroblasts.^442,443^ However, a different view was presented by Jiang et al.^444^ They suggested that GSDMD is predominantly expressed in leukocytes within the heart tissue, as opposed to other cell populations. Activation of GSDMD occurs early following AMI and is instrumental in enhancing neutrophil synthesis and recruitment to the site of myocardial damage. Elimination of GSDMD through genetic knockout or pharmacological intervention in murine models has been shown to lessen myocardial damage, decrease the size of the infarct, and enhance cardiac function and survival rates. The production and activation of bone marrow-derived neutrophils, which are GSDMD-dependent, are implicated in the detrimental immunopathology that follows AMI.^444^ The findings imply that GSDMD could represent a promising therapeutic target for the treatment of cardiovascular diseases. Zhong et al. employed a combination of virtual screening, followed by pharmacological assays, and subsequent pharmacological validation to initially identify a novel GSDMD inhibitor, termed GSDMD inhibitor Y1 (GI-Y1), which was recognized to shield cardiomyocytes from pyroptotic cell death and dysfunction, effectively inhibiting myocardial I/R injury and exerting cardioprotective effects on cardiac remodeling.^445^ GI-Y1 interacts with GSDMD and prevents the lipid-binding and pore-forming activity of GSDMD-NT by targeting the Arg7 residue, and may also attenuate mitochondrial damage by blocking the induced mitochondrial pore formation by GSDMD-NT.

Dox has been widely used in the treatment of numerous human malignancies, and has seen its broad application hindered due to side effects such as doxorubicin-induced cardiotoxicity (DIC).^446^ Dox significantly triggers GSDMD expression and cleavage in cardiac tissues. The absence of GSDMD has been observed to mitigate DIC in mice.^446,447^ Two studies suggested different mechanisms, with Ye et al. suggesting that Dox could directly engage with GSDMD, enhancing pyroptosis facilitated by GSDMD-NT, or indirectly prompt GSDMD-NT production and pyroptosis by stimulating caspase-1/11. In addition, Dox also induces mitochondrial damage and mitochondrial perforation in cardiomyocytes through Bnip3.^446^ Qu et al. illustrated that GSDMD-NT could form pores within ER, activating ER stress, which in turn, regulated the reticulophagy receptor FAM134B, interacting with the autophagy protein LC3 to instigate cardiac autophagy, accelerate cardiomyocyte apoptosis, and exacerbate DIC.^447^ Their studies confirm that GSDMD targeting and regulation may present an innovative therapeutic avenue for the prophylaxis and therapy of DIC.

Considering the above findings, it becomes evident that GSDMD exerts a crucial role in cardiovascular diseases, emerging as a promising therapeutic candidate. There is significant potential to explore the upstream transcription factors or inhibitors of GSDMD as well as the relationship between GSDMD and mitophagy and ER stress.

### Neurodegenerative diseases

Neurodegenerative disorders encompass a spectrum of neurological disorders marked by a gradual erosion of neuronal architecture and function.^448,449^ The pathogenesis is intricate, with neuroinflammation acknowledged as a crucial driver. The elucidation of pyroptosis mechanisms has drawn attention to the connection between inflammation associated with GSDMD and the pathogenesis of neurodegenerative diseases.^450,451^ Notable neurodegenerative diseases encompass Alzheimer’s disease (AD) and Parkinson’s disease (PD).^449^ Pyroptosis is implicated in the initiation of amyloid β-protein (Aβ) aggregation and neuronal death in AD, contributing to the onset and advancement of this disorder.^452^ Caspase-1 inhibition by administration of VX-765 attenuates cognitive dysfunction and neuroinflammation in an animal model. Additionally, VX-765 prevents neuronal degeneration in vitro.^453^ IL-1β is intimately involved in CNS inflammation, and pyroptosis triggered by GSDMD is upregulated in peripheral blood mononuclear cells (PBMCs) of AD patients, releasing substantial amounts of IL-1β and exacerbating AD. DSF mitigates systemic inflammation and microglia activation in mice with LPS-induced AD, lowering peripheral blood IL-1β levels and exhibiting a significant protective effect.^454^ A clinical study demonstrated that GSDMD levels were elevated in the cerebrospinal fluid of individuals with AD, potentially serving as a diagnostic biomarker.^455^ In addition, analysis of postmortem brain tissue shows that the expression of GSDMD is across a diverse array of brain cell types, including microglia, astrocytes, and neurons, and that GSDMD is cleaved not only in microglia by caspase-1, but also in astrocytes and neurons, probably through caspase-8 and caspase-4, respectively. Encountering GSDMD-NT expression in microglia and astrocytes in the immediate vicinity of Aβ deposits implies a potential influence of Aβ on the processing of GSDMD.^456^ These findings suggest that inflammasomes and GSDMD are involved in neuroinflammation in AD, but the roles and mechanisms of GSDMD in various cell types in AD remain unclear due to its complex pathogenesis. Investigating the intricacy and variability within the neuroinflammatory response in patients with AD could shed light on its functional implications.

The fundamental pathological feature of PD is the irreversible destruction of nigrostriatal dopamine neurons, a complexity of which remains elusive.^457^ GSDMD-mediated neuroinflammation, an influential contributor to PD, has lately been spotlighted. Prussian blue nanozyme (PBzyme), recently recognized as an inhibitor of pyroptosis, exhibits exceptional ROS-scavenging abilities. It inhibits the assembly of NLRP3 inflammasome, lessens activated caspase-1, downregulates GSDMD cleavage and inflammatory agent release, and impedes microglia pyroptosis in PD cellular and mouse models. Consequently, it effectively mitigates motor deficit and nigral striatal neuron impairment in a mouse model of PD.^458^ IL-1β is able to permeate the CNS parenchyma to exacerbate neuroinflammation. In a PD experimental model, peripheral myeloid cell-derived GSDMD boosts microglial immune training via a mechanism where IL-1β, crossing the blood-brain barrier, triggers microglial cell polarization, thereby amplifying neuroinflammation and neurodegeneration. Moreover, inhibition of GSDMD with DSF attenuates the bacterial infection-associated PD behavioral phenotype and dopaminergic neuron loss.^459^ These results suggest that GSDMD represents a promising new therapeutic target for PD, but further studies are needed to confirm its therapeutic potential and to elucidate the precise mechanisms underlying its action.

### Metabolic diseases

Non-alcoholic fatty liver disease (NAFLD) represents a widespread chronic liver disorder, frequently coexisting with metabolic syndrome, including hyperlipidemia, obesity, and type 2 diabetes mellitus (T2DM).^460–462^ Several reports have suggested that NLRP3 inflammasome plays a role in the pathogenesis of NAFLD.^69,463–465^ GSDMD may also contribute to its progression. Xu et al. elucidated the critical role of GSDMD in the development of non-alcoholic steatohepatitis (NASH) by mediating lipogenesis and NF-κB signaling pathway.^466^ GSDMD-NT expression is positively associated with the activity score and fibrosis in NAFLD. Compared to controls, patients with NAFLD/NASH exhibit elevated levels of hepatic GSDMD and GSDMD-NT proteins, with particularly heightened expression of GSDMD-NT observed in those with NASH. GSDMD silencing attenuates hepatic lipid accumulation, steatosis, necroinflammation, and fibrosis.^466^ These data emphasize the importance of GSDMD in the pathological progression of steatohepatitis.

IL-1β has emerged as a key driver in the exacerbation of hepatic inflammation, steatosis, injury, and fibrosis, and promotes significant production of TNF-α and monocyte chemoattractant protein-1 (MCP-1), collectively contributing to the development of NAFLD/NASH.^463,467–469^ In the mouse model, hepatic production of MCP-1, TNF-α, and IL-1β is significantly reduced in GSDMD^−/−^ mice. As NF-κB serves as a critical upstream controller of MCP-1, TNF-α, and IL-1β expression, NF-κB signaling is inhibited in GSDMD^−/−^ mice.^466^ Furthermore, GSDMD^−/−^ mice show reduced expression of genes involved in lipogenesis and enhanced expression of genes associated with lipolysis, which attenuates hepatic steatosis.^466^ It is thus known that the mechanism of inhibiting GSDMD to control disease progression includes control of cytokines secretion, NF-κB activation, and lipogenesis.

Diabetic nephropathy (DN) occurs in about 40% of diabetes patients and is the primary cause of microvascular complications and end-stage renal disease.^470^ Numerous reviews have concluded that pyroptosis participates in the onset and progression of DN,^471–474^ and to a significant degree, inhibiting pyroptosis is tantamount to mitigating the harm caused by DN, and GSDMD inhibition might be an essential target. Increased expression of TLR4 and GSDMD has been observed in both patients with DN and corresponding animal models, and suppressing the TLR4/NF-κB signaling cascade reduces the expressions of caspase-1 and GSDMD, indicating its involvement in GSDMD-associated pyroptosis in DN.^475,476^ Moreover, TLR4 can exacerbate DN tubular injury and fibrosis through the canonical pyroptosis pathway. GSDMD activation inhibits apoptosis and induces pyroptosis, potentially representing a switch mechanism between switch between TLR4-induced pyroptosis and apoptosis in the context of DN.^476^

Podocytes are an important target of injury in the early stages of DN, with their degeneration and loss being intimately linked to the manifestation of proteinuria.^477^ In diabetic mice, renal podocytes exhibit markedly heightened expression levels of caspase-11 and GSDMD-NT, concomitant with an amplified release of IL-1β and IL-18. The alterations observed in diabetic mice are mitigated by the genetic ablation of caspase-11 or GSDMD. Conversely, the silencing of caspase-4 or GSDMD via siRNA significantly reduces pyroptosis-associated modifications in vitro.^478^ Compared to WT mice, GSDMD^−/−^ mice showed reduced pyroptosis and improved kidney injury-related indices.^478,479^ In addition, glomerular endothelial cell (GECs) injury emerges as a pivotal pathological process during the early stages of DN. The non-canonical pyroptosis pathway leads to GECs damage and further aggravates the development of DN. Interference with GSDMD expression ameliorates renal pathology.^480^ These studies highlight the involvement of GSDMD in pyroptosis-induced DN, but the specific mechanisms deserve further exploration.

### Autoimmune diseases

Inflammatory bowel diseases (IBDs), including conditions such as ulcerative colitis and Crohn’s disease, represent chronic inflammatory conditions that primarily impact the gastrointestinal axis.^481^ IBDs are believed to arise from inappropriate and sustained inflammatory responses to commensal microorganisms in genetically susceptible hosts.^481,482^ Some evidence suggests the involvement of pyroptosis in IBDs. In the model of colitis induced by dextran sulfate sodium (DSS), NLRP3 inflammasome emerges as a central regulator driving intestinal inflammation. TLR4/NF-κB activation triggers NLRP3 inflammasome activation, which regulates pyroptosis of IECs and DSS-induced chronic colitis in mice.^483^ Elevated expression of epithelial-derived GSDMD has been detected in both IBD patients and experimental colitis.^271^ In colitis models, knockout of GSDMD or pharmacological inhibition of GSDMD attenuates colitis severity compared to WT mice.^271,483–486^ GSDMD is expressed in colitis IECs, and GSDMD-NT fosters IL-18 release, resulting in the loss of cupped cells and induction of colitis.^483^ The non-pyroptotic function of full-length GSDMD in guiding the generation of small extracellular vesicles (sEVs) enriched for IL-1β in IECs has been suggested as a contributory factor in intestinal inflammation, and this GSDMD-dependent non-pyroptotic role appears to be coupled with the activation of caspase-8.^271^ Moreover, inhibition of caspase-8/GSDMD-dependent pyroptosis of epithelial cells has a preventive effect on intestinal inflammation.^484^ It has been proposed that the efficacy of colitis treatment can only be optimized by concurrently disrupting both GSDMD and GSDME.^485^ Paradoxically, Ma et al. reported that macrophage-specific GSDMD deficiency, but not epithelial cell-specific GSDMD deficiency, exacerbated experimental colitis.^487^ The mechanism may be that GSDMD acts as a negative modulator within macrophages to control cGAS-dependent inflammation, thereby preventing colitis. Furthermore, GSDMB also acts as a pivotal player in the pathology of IBD. It serves as a crucial element in reestablishing epithelial barrier integrity and reducing inflammatory responses.^48^ Interestingly, its function in this context is not dependent on pyroptosis. The absence of GSDMB results in enhanced cellular adhesion, an issue that hinders the vital processes of epithelial restoration and repair, fundamental to mucosal wound healing. The underlying mechanism involves the GSDMB knockout-induced inactivation of FAK through PDGF-A-dependent pathways, leading to an upsurge in the formation of actomyosin stress fibers.^48^ FAK stands as a critical tyrosine kinase governing the transition of focal adhesions and their engagement with the cytoskeleton, whereas PDGF-A regulates FAK phosphorylation.^488–491^ These results suggest a role for GSDMs in the pathogenesis of IBD, but the functional mechanisms may differ among various cell types, warranting further investigation.

Rheumatoid arthritis (RA) is a chronic inflammatory disorder of the joints that results in erosion of cartilage and bone, culminating in disability. Excessive inflammatory cytokines contribute to the pathogenesis of RA.^492,493^ In patients with RA, synovial fluid exhibits heightened concentrations of IL-1β and IL-18, with macrophages displaying increased expression of NLRP3, caspase-1, and GSDMD-NT.^494^ Furthermore, the NLRP3 inflammasome within monocytes is triggered in patients with RA, inducing GSDMD-dependent pyroptosis and the secretion of inflammatory cytokines, including TNF-α, IL-1β, and IL-6. In turn, IL-6 exacerbates RA-derived monocyte pyroptosis.^495^ Although the understanding of NLRP3 activation in RA pathogenesis has been summarized,^496^ the function and underlying mechanisms of GSDMD in this context are still elusive.

Multiple sclerosis (MS) is a chronic, inflammatory, and demyelinating disorder of CNS, whose exact etiology is yet to be fully understood. It represents the most prevalent non-traumatic cause of disability among young adults.^497,498^ Its pathogenesis is complex, and recent evidence suggests that pyroptosis-driven inflammation may be critical in MS.^497,499–501^ GSDMD-mediated inflammasome activation and pyroptosis can occur in myelin-forming oligodendrocytes (ODCs) and microglia within the CNS of MS individuals, as well as in the experimental autoimmune encephalomyelitis (EAE) model.^500^ The administration of VX-765 to EAE models reduces pyroptosis-related protein levels within the CNS, prevents axonal damage, and improves neurological function.^500^ GSDMD^−/−^ mice are protected from EAE, with the absence of GSDMD in peripheral myeloid cells of EAE mice significantly impeding the migration of immune cells into the CNS. Consequently, this results in attenuated neuroinflammation and demyelination.^502^ Three inhibitors of GSDMD protect against EAE. DSF treatment inhibits the progression of EAE and greatly reduces clinical and histopathological scores.^502^ DMF impedes the development of EAE and reduces neuropathology and demyelination.^46^ C202-2729, a recently identified GSDMD inhibitor, significantly inhibits the aggregation of immune cells and demyelination within the spinal cord of EAE.^503^ These investigations corroborate the concept that GSDMD-induced pyroptosis serves as a determinant in the pathogenesis of MS.

Systemic lupus erythematosus (SLE) represents a complex autoimmune condition marked by the breakdown of tolerance to nucleic acids, resulting in widespread damage to peripheral organs throughout the body.^504,505^ Robust increases in the expression of GSDMD and IL-1β mRNA are observed in PBMCs from patients with SLE. DSF treatment potently inhibits serum from SLE patients-induced THP-1 pyroptosis.^506^ DSF mitigates elevated levels of serum IL-1β and GSDMD-mediated glomerular macrophage pyroptosis as well as the infiltration of inflammatory cells, proliferation of tethered cells, and structural disorders of renal tubules in pristane-induced lupus (PIL) mice.^506^ In addition, neutrophil NET promotes the development of SLE. In neutrophils, SLE serum immune complexes (ICs) and IFN-γ promote GSDMD activation through the serpinb1 and caspase-1/11 pathway. Simultaneously, these ICs induce mitochondrial stress and the extrusion of ox-mtDNA into the cytoplasm. Cytosolic ox-mtDNA binds to GSDMD-NT, promoting its oligomerization and pore formation. This sequence of events ultimately contributes to the pathogenesis of SLE through the externalization of NETs and mtDNA. The abrogation of neutrophil-specific GSDMD or the therapeutic administration of DSF substantially mitigates disease severity in the PIL mouse model.^254^ However, there are conflicting perspectives. Wang et al. suggested that GSDMD deficiency resulted in higher mortality, exacerbated renal and pulmonary inflammation, and increased production of autoantibodies within PIL mice.^507^ GSDMD negatively regulates auto-antigen production and immune dysregulation following organ damage, potentially exerting a previously unrecognized protective influence on systemic autoimmunity.

Familial Mediterranean fever (FMF) represents the prototypical monogenic autoinflammatory disorder, arising from a missense alteration in the Mefv gene that triggers the pyrin inflammasome. In a mouse FMF model, GSDMD^−/−^ mice exhibited complete protection from systemic inflammatory cytokines production, weight loss, splenomegaly, and liver damage,^258^ and pharmacological inhibition of GSDMD achieved similar protective effects.^46^

## Therapeutic targets regarding gasdermins

### GSDMD inhibitors

**Fig. 5:**
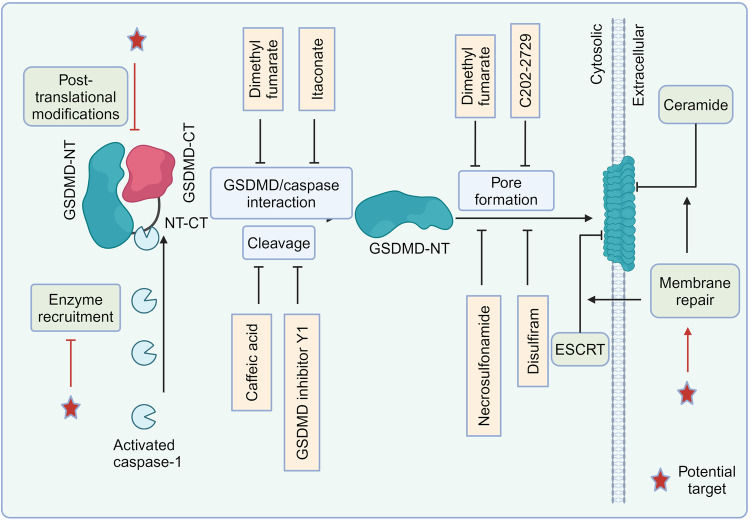
Strategies for managing GSDMD-related disorders. The activation targeting steps of GSDMD and the functioning mechanisms of existing GSDMD inhibitors are illustrated. Three potential approaches to suppress GSDMD activation are suggested: (i) preventing mutual recruitment of GSDMD and inflammatory caspases; (ii) degrading GSDMD through post-translational modifications; (iii) eliminating GSDMD pores from cell membranes to promote membrane repair. Further details can be found in the text

As previously described, the silencing or knockout of GSDMD has been demonstrated to exert a protective effect across diverse animal models of inflammatory disorders. In comparison to the selective inhibition of NLRP3 or inflammatory caspases or IL-1β, the inhibition of GSDMD may prove to be more efficacious in inflammatory diseases due to its ability to prevent the subsequent pyroptosis of all inflammasomes. Therefore, pharmacological inhibition of pyroptosis mediated by GSDMD could emerge as a promising strategy for the amelioration and control of inflammatory diseases. Here, we provide an overview of several GSDMD inhibitors (Table 4) that have different mechanisms (Fig. 5).

**Table 4:** GSDMD inhibitors

Inhibitor	IC_50_	Mechanisms of action	Off-target effects	GSDMD-related disease model
Necrosulfonamide (NSA)^44,509–511^	~10 μM	Binding directly to Cys191 of GSDMD and inhibiting the oligomerization of GSDMD-NT	Binding to Cys86 of MLKL and blocking necroptosis	LPS-induce sepsis, AD, AS, ALF
Disulfiram (DSF).^45,52,383,384,514,516–519^.	~10 μM	Modifying Cys191 of GSDMD and inhibiting the oligomerization of GSDMD-NT	Modifying Cys133 in the TLR-binding partner MD-2 and preventing LPS recognition; Inhibiting NLRP3 signaling	LPS/CLP-induced sepsis, ulcerative colitis, AS, obesity and metabolic dysfunction, SARS-CoV-2 infection, ARDS, DN, NAFLD
Dimethyl fumarate (DMF)^46,409^	<10 μM	Succinating Cys191 of GSDMD, blocking caspase-GSDMD interactions and inhibiting the oligomerization of GSDMD-NT	Succinating GSDME at Cys45; dopamine beta-hydroxylase; caspase-1; caspase-3	LPS-induced sepsis, FMF, EAE, HCC
Itaconate^320,523,531–533^	Not known	Binding to GSDMD via Cys77 and blocking caspase-GSDMD interactions	Inhibiting NLRP3 and caspase-1	ARDS, IBD, LPS-induced sepsis
C202-2729^503^	Not known	Binding directly to the GSDMD-NT and inhibiting the oligomerization of GSDMD-NT	Not known	EAE
Caffeic acid (CA)^522^	Not known	Binding directly to GSDMD and blocking GSDMD cleavage	Not known	LPS-induced sepsis
GSDMD inhibitor Y1 (GI-Y1)^445^	Not known	Binding to GSDMD via Arg7 and inhibiting the oligomerization of GSDMD-NT	Not known	Myocardial I/R injury

*AD* Alzheimer’s disease, *AS* atherosclerosis, *ALF* acute liver failure, *ARDS* acute respiratory distress syndrome, *CLP* cecum ligation and puncture, *DN* diabetic nephropathy, *EAE* experimental autoimmune encephalomyelitis, *FMF* familial Mediterranean fever, *HCC* hepatocellular carcinoma, *IBD* inflammatory bowel disease, *I/R* ischemia/reperfusion, *LPS* lipopolysaccharide

#### Necrosulfonamide

NSA is the first demonstrated inhibitor that directly targets GSDMD,^44^ although it was initially discovered to bind to Cys86 of MLKL, disrupting the disulfide bond and thereby blocking MLKL-associated necroptosis.^508^ NSA inhibits inflammasome-dependent pyroptosis by directly combining with GSDMD Cys191/Cys192 (human/mouse) and inhibiting the oligomerization of GSDMD, without affecting GSDMD cleavage or initial dimerization. A noteworthy observation is that NSA treatment is more effective than the Cys191Ala mutation, possibly because NSA activity refers to potential steric interference to oligomerization.^44^ Furthermore, follow-up studies in other models have demonstrated that NSA can also suppress the proximal events of pyroptosis, including LPS-induced gene transcription and caspase-1 activation,^45,259^ indicating that it is not entirely specific for GSDMD. The administration of NSA effectively diminishes the secretion of inflammatory cytokines and enhances survival in mice subjected to endotoxin shock.^44^ The inhibitory effects of NSA have been the subject of comprehensive investigation in various disease models, yielding promising outcomes.^509–513^ Nonetheless, the prolonged utilization of NSA, beyond its role as a research tool, is curtailed by several potential factors, including its unique mechanism of action, its capacity to inhibit necroptosis in mice, and the challenges in obtaining clinical approval.

#### Disulfiram

DSF, a therapeutic agent employed for managing chronic alcoholism through targeting aldehyde dehydrogenase (ALDH), has established a robust safety profile over numerous decades of utilization and is currently being actively considered for repurposing. Hu et al. employed a high-throughput screening approach, utilizing a fluorescent liposome leakage assay, to explore the possibility that DSF might serve as a GSDMD inhibitor, thereby preventing the onset of pyroptosis.^45^ The cellular IC50 values of DSF for inhibition of human canonical pyroptosis and mouse non-canonical pyroptosis are 7.7 ± 0.3 μM and 10.3 ± 0.5 μM, respectively. DSF potently inhibits the formation of GSDMD pores both in vitro and in vivo, as well as intracellularly, while exhibiting minimal influence on the early stages of pyroptosis. Notably, DSF does not inhibit GSDMD or IL-1β cleavage. Mechanistically, DSF modifies Cys191/Cys192 (human/mouse) to render GSDMD-NT incapable of pore-forming. Modification of other cellular targets by DSF does not result in significant clinical toxicity, and thus numerous studies have reported its potential application in inflammatory diseases. DSF has demonstrated its therapeutic utility across diverse animal disease models, including LPS/CLP-induced sepsis,^45,52,514^ ulcerative colitis,^514,515^ AS,^516^ obesity and metabolic dysfunctions,^517^ SARS-CoV-2 infection,^383^ ARDS,^384^ DN,^518^ and NAFLD.^519^ Furthermore, certain clinical trials have substantiated the anti-inflammatory attributes of DSF. In a self-controlled clinical trial (ChiCTR2100048035), DSF demonstrated an ability to modulate the human gut microbiota.^519^ Additionally, DSF potentially mitigated the occurrence and the extent of COVID-19, resulting in its evaluation in two subsequent phase II clinical trials (NCT04485130 and NCT04594343, Table 3).^520^ It is noteworthy that DSF exerts its effects not only by inhibiting GSDMD pore formation but also by altering Cys133 in MD-2, a TLR-binding partner, thereby preventing LPS recognition.^521^ Consequently, DSF robustly inhibits both extracellular and intracellular LPS-triggered innate immune responses.

#### Dimethyl fumarate

DMF, approved by the FDA for the therapeutic intervention of MS, has recently been demonstrated to be a GSDMD inhibitor, but it was previously considered not to modulate GSDMD-mediated lipid permeability.^45^ Humphries et al. demonstrated that the introduction of DMF into cells or the endogenous presence of DMF impedes the assembly of GSDMD pores and thus inhibited pyroptosis.^46^ The underlying mechanism involves DMF binding to GSDMD Cys191/Cys192 (human/mouse) and preventing caspase-1/GSDMD interaction rather than caspase-1 cleavage, ultimately blocking GSDMD cleavage, oligomerization, and cell death. DMF treatment also modifies other GSDMD cys residues, suggesting that succination may have additional off-target effects. In addition to inhibiting GSDMD, DMF also succinates GSDME at the Cys45 site to block GSDME cleavage and GSDME-dependent pyroptosis. DMF has been used across a spectrum of animal models of inflammatory diseases to reduce the severity, including LPS-induced sepsis,^46,522^ FMF,^46^ EAE,^46^ and HCC.^409^

#### Itaconate

Itaconate emerges as a distinct regulatory metabolite in myeloid cells following TLR activation, functioning as an intrinsic modulator that curtails the progression of inflammasome activation and pyroptotic cell death. The post-translational modification function of endogenous itaconate on GSDMD was elucidated by Bambouskova et al. Itaconate blocks caspase-1 activation and GSDMD cleavage and enhances cellular tolerance to prolonged LPS stimulation.^320^ Mechanistically, itaconate binds to GSDMD via Cys77, which has previously been shown to be essential for the oligomerization process and may interfere with caspase/GSDMD interactions, thereby inhibiting pyroptosis. However, research has also demonstrated that itaconate inhibited NLRP3^523^ and caspase-1,^320^ suggesting that its inhibitory effects on pyroptosis are non-specific and may not solely target GSDMD.

#### C202-2729

C202-2729, an unreported small molecule, has recently been recognized as an inhibitor of pyroptosis. A virtual screen of ChemDiv compounds conducted by Cao et al. revealed that C202-2729 potently inhibits inflammation, manifesting robust anti-inflammatory activity in mouse models of endotoxin shock and EAE.^503^ C202-2729 does not affect either the cleavage of GSDMD or the initiation of inflammasome activation upstream. Instead, it physically associates with the GSDMD-NT, preventing its movement to the PM and the subsequent formation of pores, thereby inhibiting the release of mature IL-1β. The proposed mechanism suggests that C202-2729 could engage with the GSDMD-NT through interactions with Tyr-54 and Lys-235, although this has not been experimentally confirmed.

#### Caffeic acid

Considering the effectiveness and relative safety of natural compounds, Liu et al. explored the effects of natural compounds on pyroptosis. They found that caffeic acid (CA) inhibited canonical pyroptosis and non-canonical pyroptosis, contributing to the mitigation of LPS-induced sepsis in mice.^522^ The inhibitory effect of CA on pyroptosis is not contingent upon its influence on cellular lipid peroxidation, mitochondrial functionality, or the expression of genes pertinent to pyroptosis. Mechanistically, CA prevents pyroptosis by directly binding to and blocking the processing of GSDMD, thereby diminishing the formation of GSDMD pores and the subsequent release of cellular contents. CA interacts with GSDMD-NT, possibly through the formation of hydrogen bonds with key residues, such as Asp22, Lys52, Tyr55, and Arg54.

#### GI-Y1

GSDMD inhibitor Y1 (GI-Y1), named by Zhong et al., was screened for pyroptosis inhibition from a library of seven commercial compounds using virtual and pharmacological screening and subsequent in vitro and in vivo pharmacological validation.^445^ GI-Y1 demonstrates selectivity for GSDMD, inhibiting GSDMD cleavage and membrane binding of GSDMD-NT, without affecting caspase-1, caspase-11, or GSDME activation. By targeting Arg7 residues, GI-Y1 inhibits the interaction between PM and GSDMD-NT and decreases the secretion of inflammatory cytokines, thereby increasing the sepsis survival rate and providing protection against myocardial I/R injury and cardiac remodeling in mice. Furthermore, GSDMD-NT interacts with mitochondria and causes mitochondrial permeabilization, leading to mitochondrial oxidative stress,^524^ and GI-Y1 effectively inhibit mitochondrial binding and mitochondrial damage by GSDMD-NT.

The discovery of the above inhibitors and animal studies indicate that blocking pyroptosis associated with GSDMD can effectively improve diverse disease models, thus corroborating GSDMD as a prospective drug target. Since 2018, at least three direct pharmacological inhibitors (NSA, DSF, DMF) have been demonstrated to suppress pyroptosis and subsequent inflammation via modulating GSDMD cleavage or interfering with GSDMD pore formation, which are predominantly mediated by covalent modification of residue Cys191. Nonetheless, these three extensively studied molecules exhibit a lack of specificity, as numerous proteins have active sulfhydryl groups in vivo. Consequently, covalent modification of sulfhydryl groups on various targets, in addition to upstream caspases and GSDMD, could potentially result in deleterious side effects. This limitation could potentially curtail their future application. It is likely that other mechanisms for inhibiting the binding of GSDMD and inflammatory caspases, post-translational modifications to degrade GSDMD, and modulation of GSDMD pore formation are plausible avenues for discovery, which could yield attractive drug targets (Fig. 5).

Furthermore, the development of GSDMD inhibitors might be encumbered by several drawbacks: (i) GSDMD typically operates as a non-singular pivotal signaling node in the inflammasome activation pathway or pyroptosis. As previously discussed, GSDMD possesses non-pyroptosis functions, such as ion and cytokine channels, which concurrently contribute significantly to disease progression. Therefore, experimental inhibition of GSDMD should take these factors into consideration; (ii) GSDME has been demonstrated to encompass numerous functions similar to those of GSDMD, suggesting that only inhibition of the associated effector cell GSDMD might not yield optimal results. Consistent with the requirement of both GSDMD and GSDME for the release of IL-1β by NLRP3 and NLRP1 inflammasomes,^525^ the concurrent inhibition of these two proteins is essential to optimize therapeutic efficacy in the treatment of colitis.^485^ When investigating GSDMD inhibitors, it is crucial to account for the possibility of other GSDMs acting as reservoirs of GSDMD activity; (iii) The current investigation of GSDMD inhibitors is overwhelmingly centered on a single pyroptosis pathway involving caspases and GSDMD, which, admittedly, represents the most straightforward and efficient strategy. However, emerging studies suggest robust interplay among pyroptosis, apoptosis, necroptosis, and other types of PCD, and in particular, the concept of PANoptosis has been proposed.^526^ GSDMD inhibitors deserve further scrutiny in the overall view of cell death.

### Manipulating intracellular gasdermins expression

Modulating the expression of intracellular GSDMs is gaining traction as a prospective therapeutic approach for treating diseases. Wang et al. have shown that the combination of phenylalanine trifluoroborate (Phe-BF_3_) with nanoparticles is able to preferentially deliver GSDMA3 to tumor cells, triggering pyroptosis via Phe-BF_3_-mediated desilylation.^527^ In experimental settings, the activation of pyroptosis in a subset of tumor cells, as low as 15%, has been shown to be efficacious in curtailing tumor growth, which is correlated with enhanced anti-tumor immune responses, and it could potentially synergize with immune checkpoint blockade therapy.^527^ Zhong et al. developed nanoliposomes encapsulating GSDME plasmids and manganese carbonyl (MnCO), which upon entry into tumor cells, facilitated CO/caspase-3/GSDME-mediated pyroptosis.^528^ Additionally, Mn^2+^ can activate the STING signaling pathway, potentiating the therapeutic effects of tumor immunotherapy when combined with inflammation induced by pyroptosis.^528^ These studies lay the groundwork for targeted GSDM-based cancer therapies. However, it is crucial to exercise caution to prevent the onset of hyper-pyroptosis, which may pose toxicity to healthy cells or lead to an uncontrolled release of cytokines and systemic consequences. This approach holds potential for broader applications in the treatment of inflammatory disorders.

## Conclusions and future perspectives

Ever since the groundbreaking identification of GSDMD as a target for inflammatory caspases, a growing body of research has fueled interest and ignited investigation into GSDMs. As the crucial executor of diverse pyroptosis pathways, GSDMs have gained prominence in various inflammatory diseases, including sepsis, virus infections, AS, T2DM, NASH, and several neurodegenerative diseases, including AD. Over the past few years, research on GSDMD has contributed invaluable insights: (i) In addition to the inflammatory caspases, apoptotic caspase-8, as well as cathepsin G and NE, are implicated in the cleavage of GSDMD and the subsequent formation of pores; (ii) Interplay between apoptotic and pyroptotic pathways suggests a sophisticated network of interactions within the cell death machinery. For example, caspase-3 and caspase-7 independently cleave GSDMD at the Asp residue, generating an inactive NT fragment (p45) that serves to specifically inhibit the activation of GSDMD; (iii) The generation of GSDMD pores is not invariably predictive of cytolysis. ESCRT-III dynamically repairs GSDMD pores, delaying or preventing the pyroptotic process. Moreover, GSDMD can serve as a channel for ions or inflammatory cytokines but is not accompanied by cell lysis; (iv) GSDMD inhibitors, such as NSA, DSF, and DMF, have shown promising outcomes in mouse models of inflammatory diseases, and an array of novel inhibitors is being identified.

However, despite extensive research, there are still numerous questions surrounding GSDMD that need to be addressed: (i) The destiny of GSDMD exhibits variability across diverse cell types and in various physiological or pathological contexts. For instance, macrophage GSDMD exacerbates inflammatory progression, and augments mortality in sepsis, yet GSDMD is emerging as a critical player in the physiological role of epithelial cells in maintaining intestinal mucosal homeostasis. The elucidation of additional physiological roles for GSDMD remains an open question; (ii) At present, although proteases such as apoptotic caspases, NE, and cathepsin G are known to cleave GSDMD, the mechanism by which they are recognized and cleaved remains obscure; (iii) How the non-pyroptosis function of GSDMD is realized remains not yet fully understood. An associated issue is whether the modulation of GSDMD activation results in lysogenic cell death or an excessive response devoid of accompanying cell mortality; (iiii) More recently, GSDMD inhibitors have been reported, yet they suffer from a lack of specificity, and the unknown risk of toxicological consequences limits their further clinical utility. The identification of GSDMD inhibitors and their implementation into the treatment of relevant inflammatory conditions presents an ongoing challenge. It remains unclear whether studies of the molecular mechanisms of pore formation and cell lysis, which follow pyroptosis, will yield novel therapeutic approaches, including the targeting of NINJ1.

In summary, pyroptosis, as a manner of PCD, is pertinent to a variety of inflammatory conditions. With GSDMs representing a burgeoning area of study, more specific functions of GSDMs in inflammation and corresponding diseases remain to be illuminated. Consequently, additional animal experiments and clinical trials are requisite to further investigate and corroborate the role and underlying mechanism of GSDMs. Moreover, it would be both advantageous and enlightening to take into account more intricate factors in the pursuit of discovering and developing more potent GSDM inhibitors.
